# Supplementary material for: Coccolithophore assemblage composition during the Greenland Interstadial–Stadial 20 transition and their response to the Youngest Toba Tuff (YTT) supereruption ∼74,000 years ago in the northeastern Arabian Sea
Source: PLoS One. 2024 Sep 25;19(9):e0310041. doi: 10.1371/journal.pone.0310041 (PMC11423970; doi:10.1371/journal.pone.0310041)
Supplement: S1 Text — (DOCX) [file pone.0310041.s001.docx]

**S1 Text. Additional frequency analyses using linearly interpolated data.**

In addition to the “redfit” function, several other methods of frequency analyses were performed on the data set such as multitaper spectral analysis (MTM; [1]) and wavelet analysis [2,3]. To perform these analyses, the relative abundance data were linearly interpolated at 7.9-year time steps (i.e., mean time interval). We then used the Acycle software [4] to conduct the frequency analyses. We used a time-bandwidth product of 2 for the MTM analysis. Lastly, we implemented ensemble empirical mode decomposition (EEMD) analysis [5]—a data-adaptive approach that does not require stationarity or linearity in extracting intrinsic oscillations (i.e., intrinsic mode functions) in the data. The EEMD analysis was done using the “rlibeemd” package in R using the default settings [6].

The MTM spectral analysis shows broadly comparable results with the “redfit” analysis (S3 Fig) although wavelet spectra indicate that these frequencies are significant in a particular time frame (S4 Fig). On the other hand, signal decomposition using the EEMD reveals a ~60–70-year cyclicity in *G. oceanica* and the small placoliths that seem to be anticorrelated (S5 and S6 Figs). However, such a cyclicity was not detected from “redfit” (Fig 3), MTM (S3 Fig), and wavelet analyses (S4 Fig).

**References from the Supporting Information**

1. Thomson DJ. Spectrum estimation and harmonic analysis. Proc IEEE. 1982;70: 1055–1096. doi:10.1109/PROC.1982.12433

2. Torrence C, Compo GP. A Practical Guide to Wavelet Analysis. Bull Am Meteorol Soc. 1998;79: 61–78. doi:10.1175/1520-0477(1998)079<0061:APGTWA>2.0.CO;2

3. Grinsted A, Moore JC, Jevrejeva S. Application of the cross wavelet transform and wavelet coherence to geophysical time series. Nonlinear Process Geophys. 2004;11: 561–566. doi:10.5194/npg-11-561-2004

4. Li M, Hinnov L, Kump L. Acycle: Time-series analysis software for paleoclimate research and education. Comput Geosci. 2019;127: 12–22. doi:10.1016/j.cageo.2019.02.011

5. Wu Z, Huang NE. Ensemble Empirical Mode Decomposition: A noise-assisted data analysis method. Adv Adapt Data Anal. 2009;01: 1–41. doi:10.1142/S1793536909000047

6. Luukko PJJ, Helske J, Räsänen E. Introducing libeemd: a program package for performing the ensemble empirical mode decomposition. Comput Stat. 2016;31: 545–557. doi:10.1007/s00180-015-0603-9
