# Supplementary figures and images for: Coccolithophore assemblage composition during the Greenland Interstadial–Stadial 20 transition and their response to the Youngest Toba Tuff (YTT) supereruption ∼74,000 years ago in the northeastern Arabian Sea
Source: PLoS One. 2024 Sep 25;19(9):e0310041. doi: 10.1371/journal.pone.0310041 (PMC11423970; doi:10.1371/journal.pone.0310041)

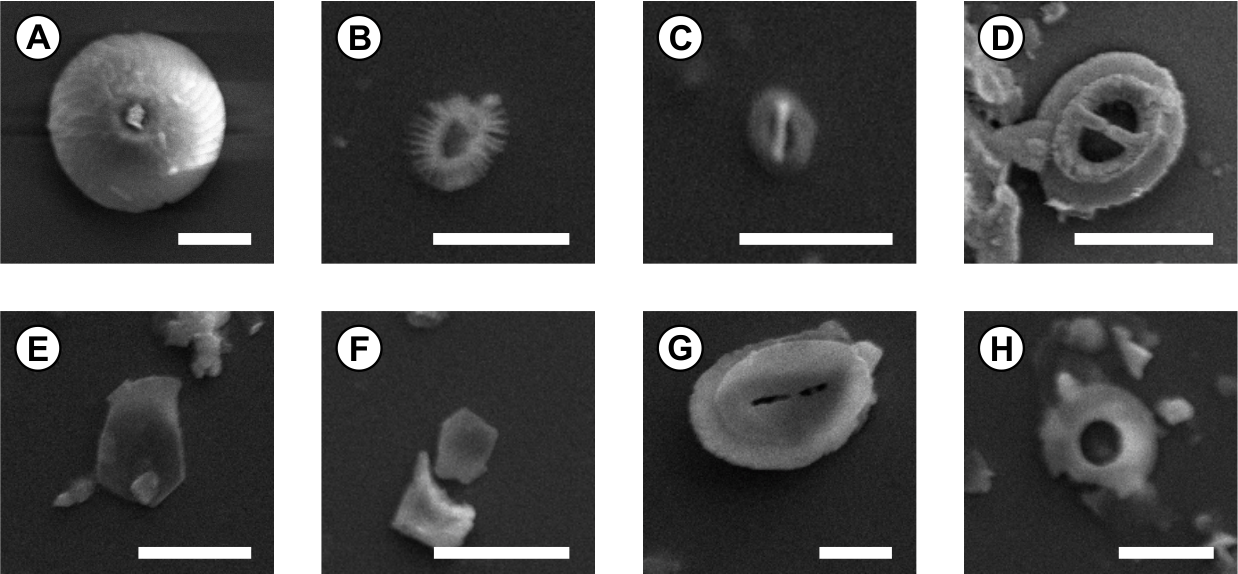

Supplement: S1 Fig — In all panels, the scale bar is 5 μm. (A) Calcidiscus leptoporus, 1842.7 cm core depth. (B) Emiliania huxleyi, 1842.7 cm core depth. (C) Gephyrocapsa ericsonii, 1843.1 cm core depth. (D) G. oceanica, 1843.7 cm core depth. (E) Florisphaera profunda var. elongata, 1843.1 cm core depth. (F) F. profunda var. profunda, 1840.7 cm core depth. (G) Helicosphaera carteri, 1843.1 cm core depth. (H) Umbilicosphaera sibogae, 1842.2 cm core depth. (TIF) [file pone.0310041.s002.tif]

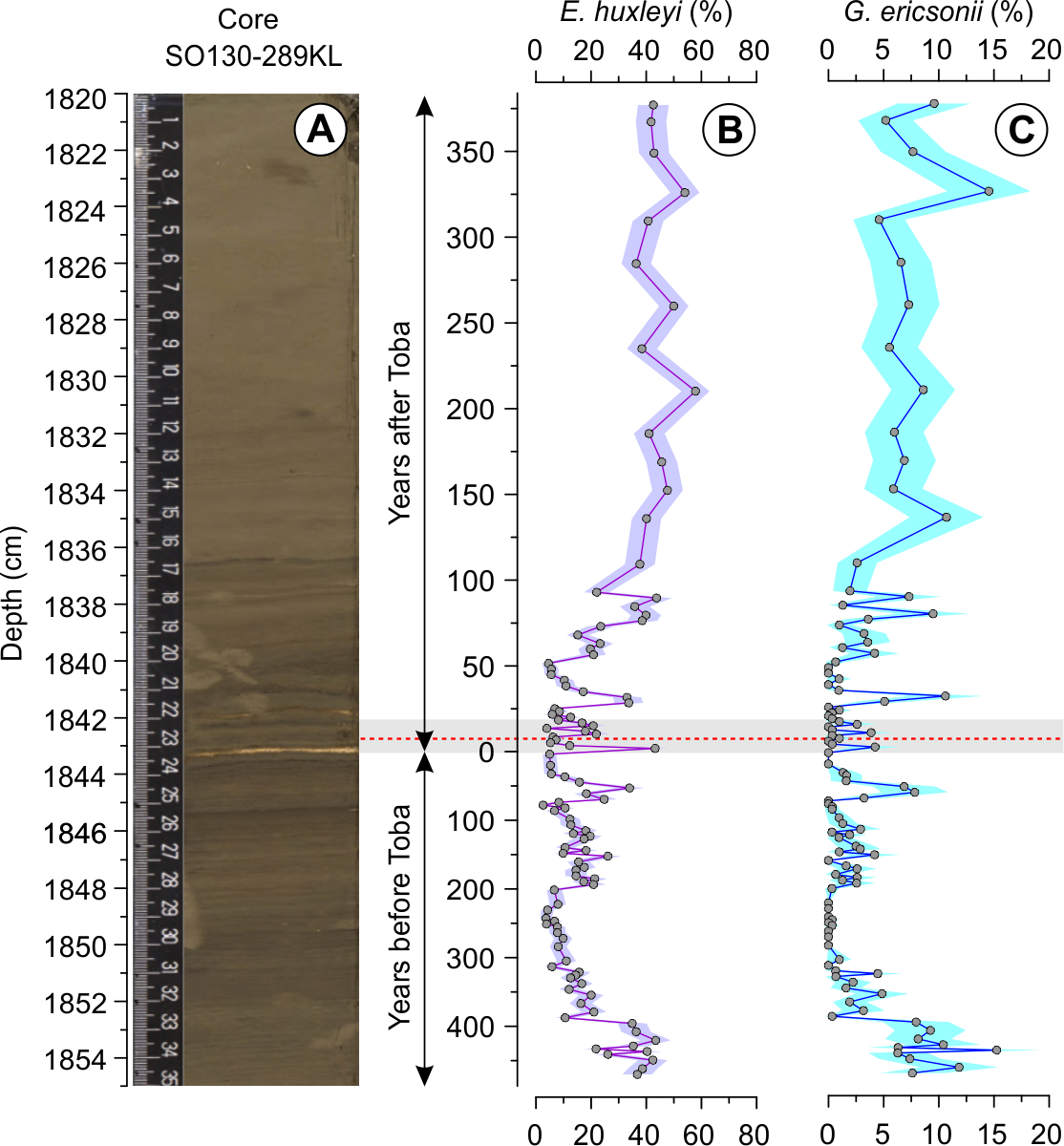

Supplement: S2 Fig — (A) Sediment core scan from Deplazes et al. [49] showing clearly visible YTT layers. (B) E. huxleyi. (C) G. ericsonii. Color bands for each species demarcate the 95% confidence interval for the relative abundances. The gray band shows the miniature slump fold enclosed by two ash layers from the YTT eruption, with the axial trace delineated by the red dotted line. (TIF) [file pone.0310041.s003.tif]

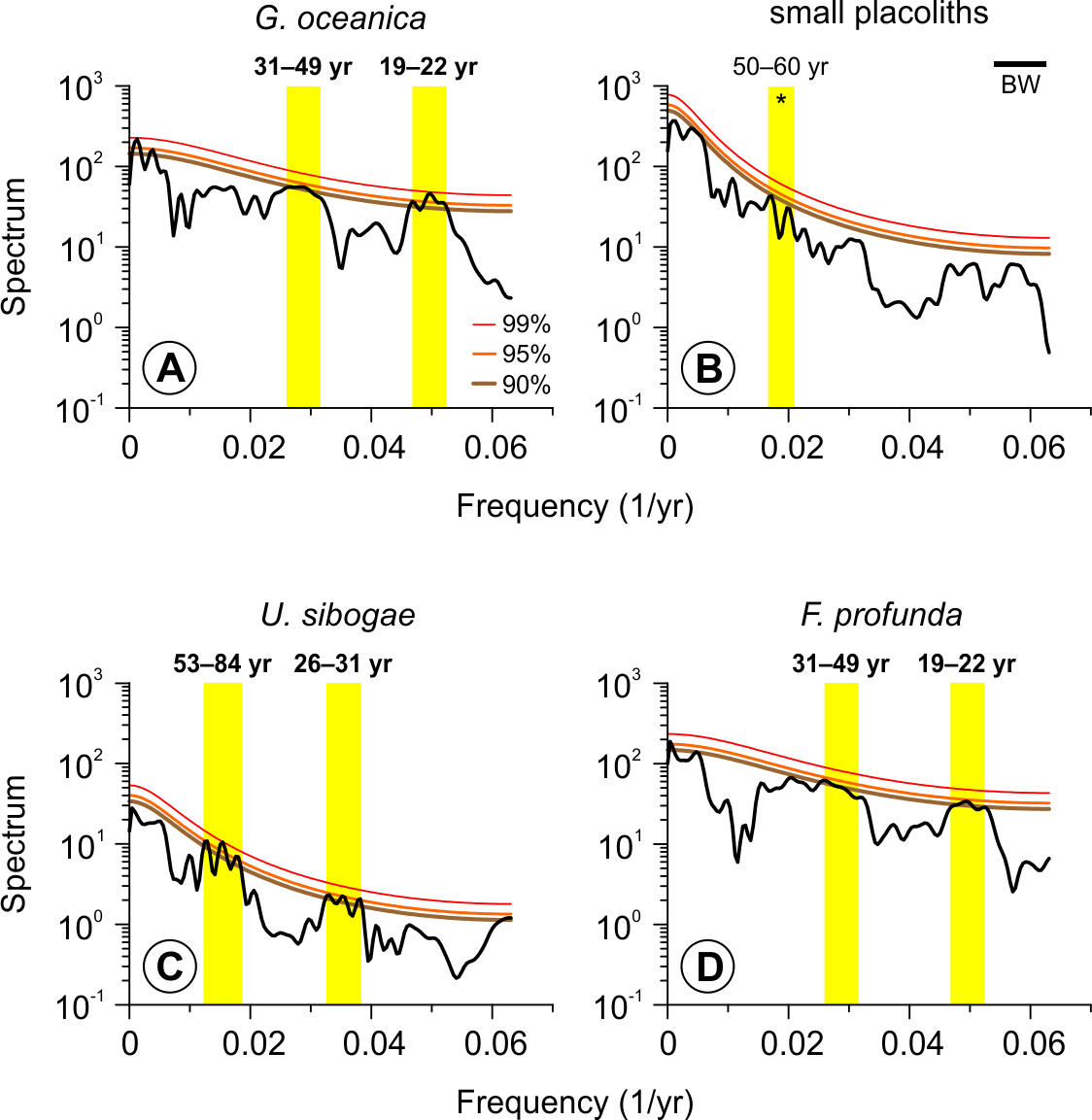

Supplement: S3 Fig — (A) G. oceanica. (B) small placoliths consisting of E. huxleyi and G. ericsonii. (C) U. sibogae. (D) F. profunda. The black line shows the spectrum for each species. Brown, orange, and red lines represent the 90%, 95%, and 99% confidence limits, respectively. The yellow rectangles highlight bands exceeding the 90% confidence limit. Yellow rectangles with asterisks show bands that are very close to the 90% confidence limit. The horizontal line in (B) shows the bandwidth (BW) resolution. (TIF) [file pone.0310041.s004.tif]

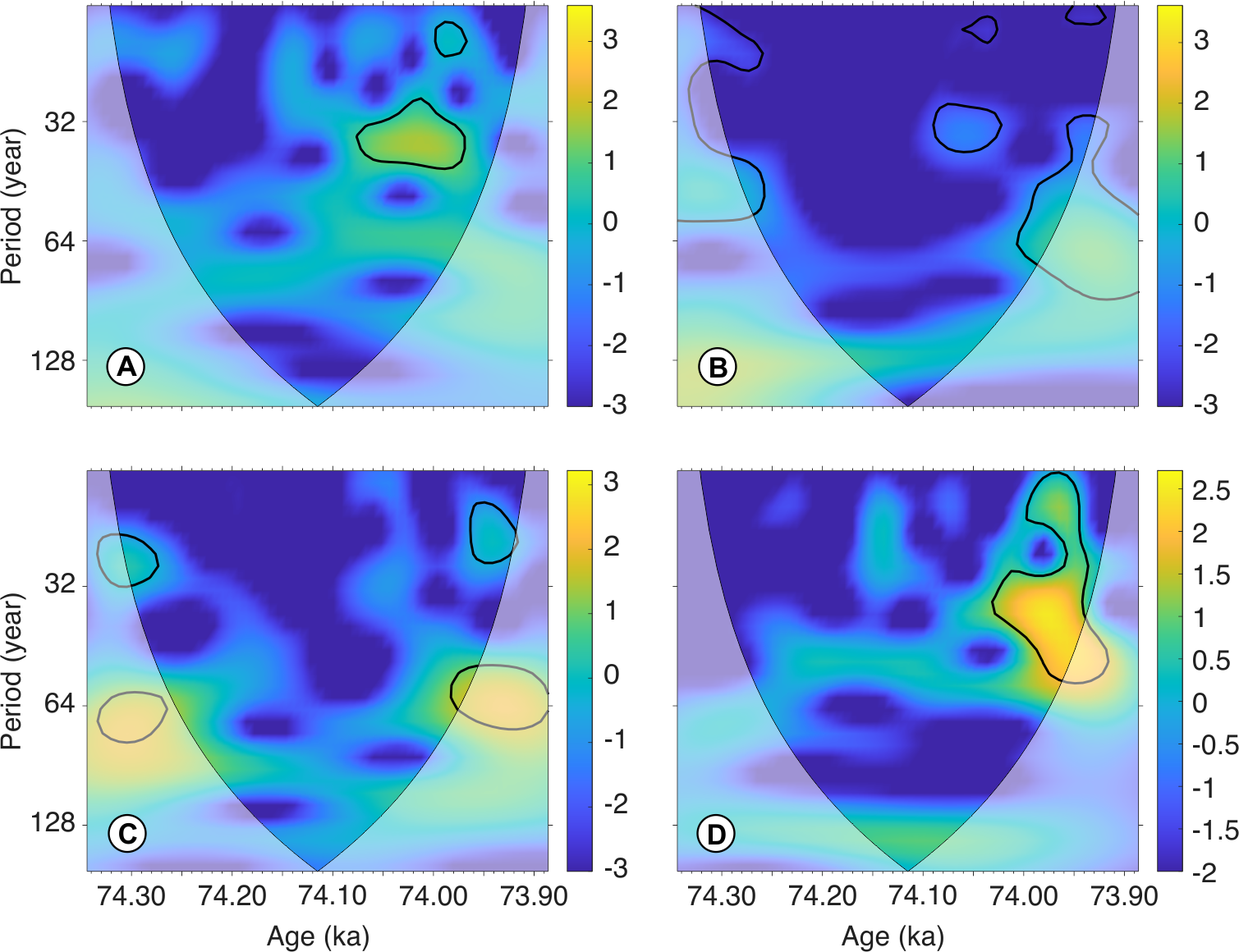

Supplement: S4 Fig — A. G. oceanica. B. small placoliths consisting of E. huxleyi and G. ericsonii. C. U. sibogae. D. F. profunda. The heavy black contour lines denote the 95% significance level. Gray shaded regions show the cone of influence, where edge effects become important and thus should be interpreted with caution (i.e., the calculation of the spectral variance lies outside of the data boundaries). (TIF) [file pone.0310041.s005.tif]

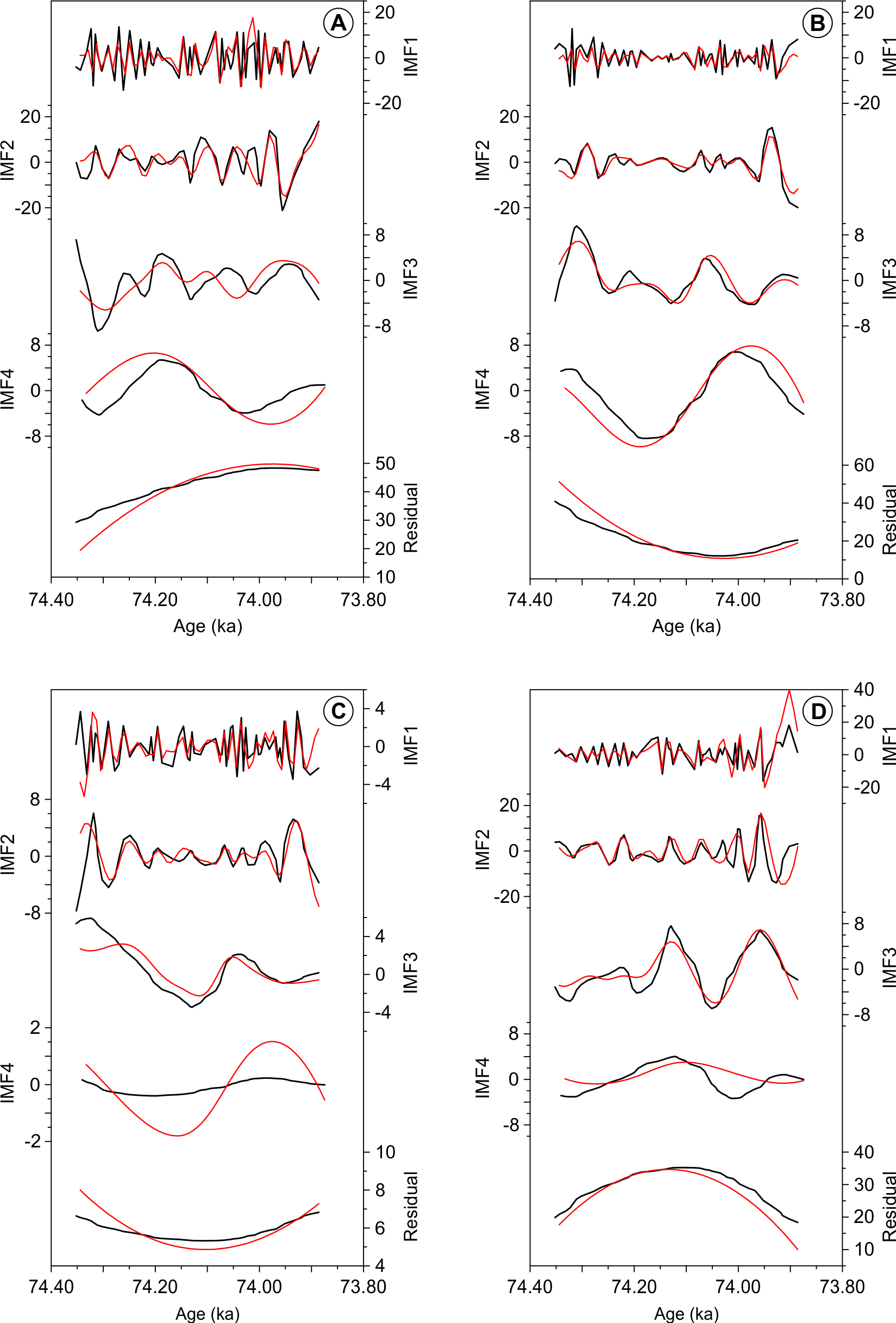

Supplement: S5 Fig — In all panels, four intrinsic mode functions (IMF) show oscillations inherent to the relative abundance data, along with a residual. The EEMD analysis was done using both raw (black lines) and interpolated (red lines) relative abundance data. A. G. oceanica. B. small placoliths consisting of E. huxleyi and G. ericsonii. C. U. sibogae. D. F. profunda. (TIF) [file pone.0310041.s006.tif]

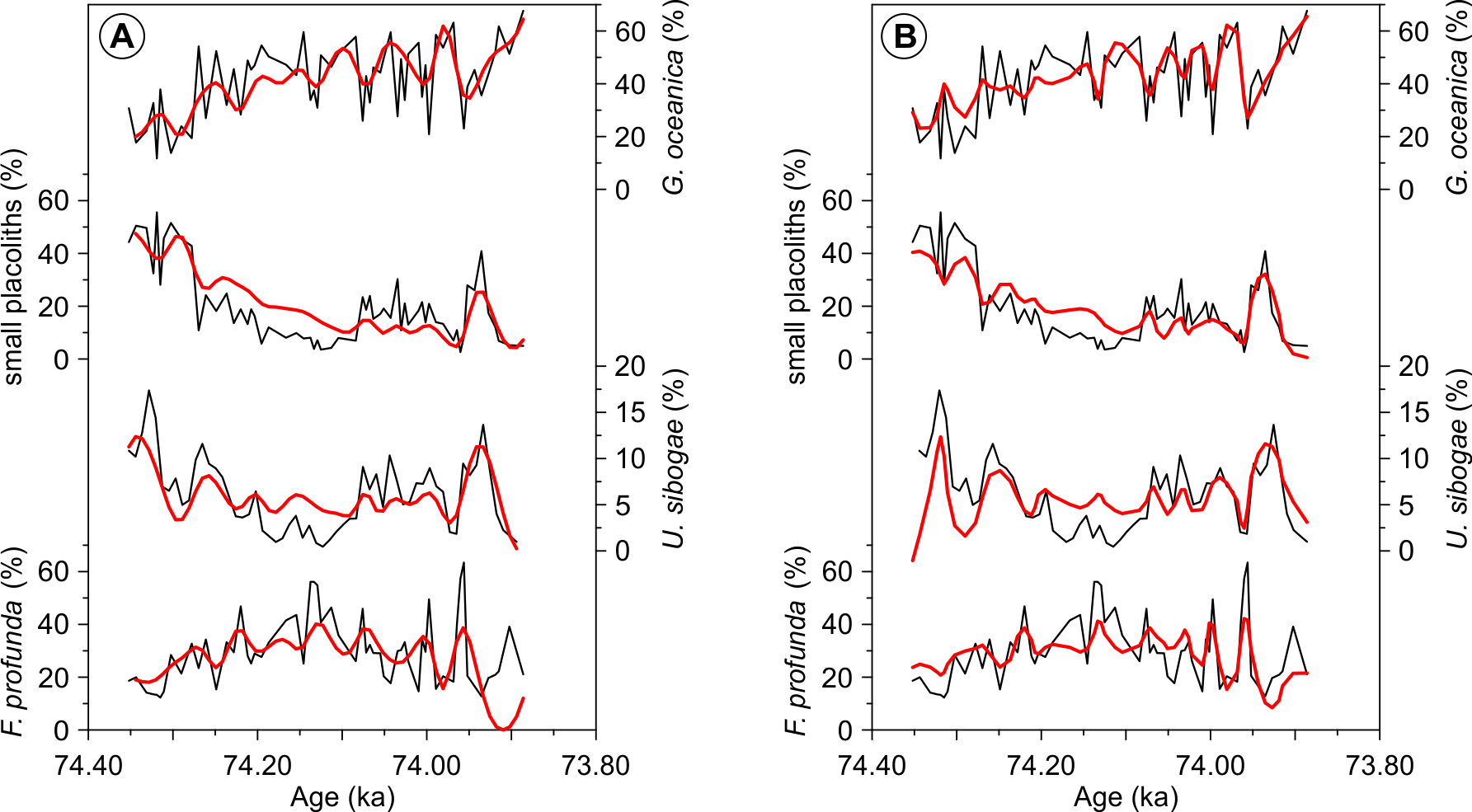

Supplement: S6 Fig — In all panels, the relative abundance data (black lines) are plotted with the residual + IMF2 (red lines) for both interpolated (A) and raw (B) datasets. (TIF) [file pone.0310041.s007.tif]

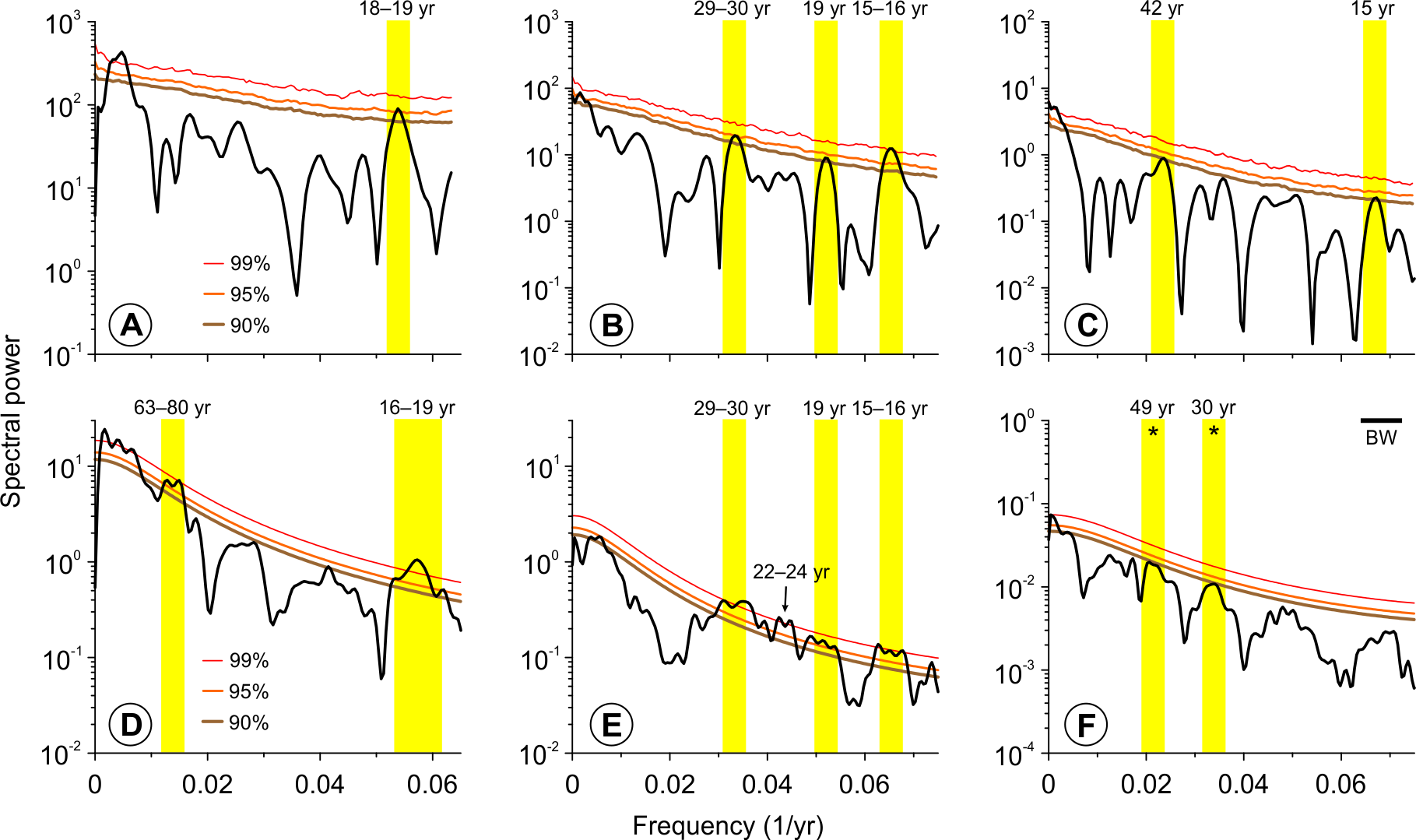

Supplement: S7 Fig — (A) and (D): L* reflectance of core SO130-289KL [38]. (B) and €: North Greenland Ice Core Project (NGRIP) δ18O record [88]. (C) and (F): The YK07 speleothem stable oxygen isotope (δ18O) record from southwest China [27]. The black line shows the spectrum for each proxy record. Brown, orange, and red lines represent the 90%, 95%, and 99% confidence limits, respectively. The yellow rectangles highlight bands exceeding the 90% confidence limit. Yellow rectangles with asterisks show bands that are very close to the 90% confidence limit. The horizontal line in (F) shows the bandwidth (BW) resolution for the multitaper analysis. (TIF) [file pone.0310041.s008.tif]
