# Supplementary material for: Coccolithophore assemblage composition during the Greenland Interstadial–Stadial 20 transition and their response to the Youngest Toba Tuff (YTT) supereruption ∼74,000 years ago in the northeastern Arabian Sea
Source: PLoS One. 2024 Sep 25;19(9):e0310041. doi: 10.1371/journal.pone.0310041 (PMC11423970; doi:10.1371/journal.pone.0310041)
Supplement: Attachment — Submitted filename: PONE-D-24-25299+MC.pdf [file pone.0310041.s010.pdf]

PLOS ONE

Coccolithophore assemblage composition during the Greenland Interstadial—Stadial 20 transition and their response to the Youngest Toba Tuff (YTT) supereruption ~74,000 years ago in the northeastern Arabian Sea

--Manuscript Draft--

**Manuscript Number:**	PONE-D-24-25299
**Article Type:**	Research Article
**Full Title:**	Coccolithophore assemblage composition during the Greenland Interstadial–Stadial 20 transition and their response to the Youngest Toba Tuff (YTT) supereruption ~74,000 years ago in the northeastern Arabian Sea
**Short Title:**	Coccolithophore assemblage response to abrupt climate change
**Corresponding Author:**	Jose Dominick Santos Guballa<br>University of Toronto<br>Toronto, Ontario CANADA
**Keywords:**	coccolithophores; Arabian Sea; core SO130-289KL; Youngest Toba Tuff eruption; Greenland Interstadial 20; Greenland Stadial 20
**Abstract:**	Here we present a new, 850-year long nearly annually resolved coccolithophore record in core SO130-289KL from the northeastern Arabian Sea that spans the Greenland Interstadial 20–Greenland Stadial 20 transition including the timing of the ~74 ka Youngest Toba Tuff (YTT) supereruption. During the warm interstadial, the coccolithophore assemblage is dominated by Gephyrocapsa oceanica (41%) and Florisphaera profunda (30%) and changes to small placolith-dominated (Emiliania huxleyi + G. ericsonii) (52%) in the succeeding cold stadial. However, the oldest samples of the study interval seem to show a stadial-like coccolithophore assemblage. Spectral analysis revealed a bidecadal cycle imprinted in the coccolithophore record during the interstadial that was also independently reported in other terrestrial and marine proxies. Immediately after the YTT eruption, the small placoliths increased by 42% from 5% right below the YTT layer to 47% ~1 mm above the YTT layer, while G. oceanica and Helicosphaera carteri increased and F. profunda decreased within the ~1.15 cm layer representing about 8–19 years.<br>Subsequently, the coccolithophore assemblage returned to a composition similar to the warm interstadial period before abruptly changing to a stadial composition dominated by small placoliths about 100–150 years after the YTT eruption. Therefore, the YTT eruption had a significant impact on the overall coccolithophore assemblage but appears to have not caused the climate transition from interstadial to stadial conditions, supporting proxy and modeling data. However, the overall mechanism driving the observed changes and cyclicities remains unknown but might be related to rapid atmospheric teleconnections of North Atlantic climate variability to the low latitudes.
**Order of Authors:**	Jose Dominick Guballa
	Jörg Bollmann
	Katherine Schmidt
	Andreas Lückge
**Additional Information:**	
**Question**	**Response**
**Financial Disclosure**<br><br>Enter a financial disclosure statement that describes the sources of funding for the work included in this submission. Review the submission guidelines for detailed requirements. View published research	Yes

Powered by Editorial Manager® and ProduXion Manager® from Aries Systems Corporation

articles from *PLOS ONE* for specific examples.<br><br>This statement is required for submission and **will appear in the published article** if the submission is accepted. Please make sure it is accurate.<br><br>**Funded studies**<br>Enter a statement with the following details:<br>• Initials of the authors who received each award<br>• Grant numbers awarded to each author<br>• The full name of each funder<br>• URL of each funder website<br>• Did the sponsors or funders play any role in the study design, data collection and analysis, decision to publish, or preparation of the manuscript?<br><br>Did you receive funding for this work?	
Please add funding details.<br>as follow-up to "**Financial Disclosure**<br><br>Enter a financial disclosure statement that describes the sources of funding for the work included in this submission. Review the submission guidelines for detailed requirements. View published research articles from *PLOS ONE* for specific examples.<br><br>This statement is required for submission and **will appear in the published article** if the submission is accepted. Please make sure it is accurate.	This work was funded by NSERC through J. B.'s NSERC Discovery Grant [http://www.nserc-crsng.gc.ca]. NSERC had no role in the study design, data collection and analysis, decision to publish, or preparation of the manuscript.

**Funded studies**<br>Enter a statement with the following details:<br>• Initials of the authors who received each award<br>• Grant numbers awarded to each author<br>• The full name of each funder<br>• URL of each funder website<br>• Did the sponsors or funders play any role in the study design, data collection and analysis, decision to publish, or preparation of the manuscript?<br><br>Did you receive funding for this work?"	
Please select the country of your main research funder (please select carefully as in some cases this is used in fee calculation).<br>as follow-up to "**Financial Disclosure**<br><br>Enter a financial disclosure statement that describes the sources of funding for the work included in this submission. Review the submission guidelines for detailed requirements. View published research articles from *PLOS ONE* for specific examples.<br><br>This statement is required for submission and **will appear in the published article** if the submission is accepted. Please make sure it is accurate.<br><br>**Funded studies**<br>Enter a statement with the following details:<br>• Initials of the authors who received each award<br>• Grant numbers awarded to each author<br>• The full name of each funder<br>• URL of each funder website<br>• Did the sponsors or funders play any role in the study design, data collection and analysis, decision to publish, or preparation of the manuscript?<br><br>Did you receive funding for this work?"	CANADA - CA
**Competing Interests**	The authors have declared that no competing interests exist.

Powered by Editorial Manager® and ProduXion Manager® from Aries Systems Corporation

Use the instructions below to enter a competing interest statement for this submission. On behalf of all authors, disclose any competing interests that could be perceived to bias this work—acknowledging all financial support and any other relevant financial or non-financial competing interests.<br><br>This statement is required for submission and **will appear in the published article** if the submission is accepted. Please make sure it is accurate and that any funding sources listed in your Funding Information later in the submission form are also declared in your Financial Disclosure statement.<br><br>View published research articles from *PLOS ONE* for specific examples.<br><br>**NO authors have competing interests**<br><br>Enter: *The authors have declared that no competing interests exist.*<br><br>**Authors with competing interests**<br><br>Enter competing interest details beginning with this statement:<br><br>*I have read the journal's policy and the authors of this manuscript have the following competing interests: [insert competing interests here]*<br><br>* typeset	
**Ethics Statement**<br><br>Enter an ethics statement for this submission. This statement is required if the study involved:<br><br>• Human participants<br>• Human specimens or tissue<br>• Vertebrate animals or cephalopods<br>• Vertebrate embryos or tissues	N/A

Powered by Editorial Manager® and ProduXion Manager® from Aries Systems Corporation

• Field research<br><br>Write "N/A" if the submission does not require an ethics statement.<br><br>General guidance is provided below. Consult the submission guidelines for detailed instructions. **Make sure that all information entered here is included in the Methods section of the manuscript.**	

**Format for specific study types**<br><br>**Human Subject Research (involving human participants and/or tissue)**<br>• Give the name of the institutional review board or ethics committee that approved the study<br>• Include the approval number and/or a statement indicating approval of this research<br>• Indicate the form of consent obtained (written/oral) or the reason that consent was not obtained (e.g. the data were analyzed anonymously)<br><br>**Animal Research (involving vertebrate animals, embryos or tissues)**<br>• Provide the name of the Institutional Animal Care and Use Committee (IACUC) or other relevant ethics board that reviewed the study protocol, and indicate whether they approved this research or granted a formal waiver of ethical approval<br>• Include an approval number if one was obtained<br>• If the study involved *non-human primates*, add *additional details* about animal welfare and steps taken to ameliorate suffering<br>• If anesthesia, euthanasia, or any kind of animal sacrifice is part of the study, include briefly which substances and/or methods were applied<br><br>**Field Research**<br><br>Include the following details if this study involves the collection of plant, animal, or other materials from a natural setting:<br>• Field permit number<br>• Name of the institution or relevant body that granted permission	
**Data Availability**<br><br>Authors are required to make all data underlying the findings described fully available, without restriction, and from the time of publication. PLOS allows rare exceptions to address legal and ethical concerns. See the PLOS Data Policy and FAQ for detailed information.	Yes - all data are fully available without restriction

A Data Availability Statement describing where the data can be found is required at submission. Your answers to this question constitute the Data Availability Statement and **will be published in the article**, if accepted.<br><br>**Important:** Stating 'data available on request from the author' is not sufficient. If your data are only available upon request, select 'No' for the first question and explain your exceptional situation in the text box.<br><br>Do the authors confirm that all data underlying the findings described in their manuscript are fully available without restriction?	
**Describe where the data may be found in full sentences. If you are copying our sample text, replace any instances of XXX with the appropriate details.**<br><br>• If the data are **held or will be held in a public repository**, include URLs, accession numbers or DOIs. If this information will only be available after acceptance, indicate this by ticking the box below. For example: *All XXX files are available from the XXX database (accession number(s) XXX, XXX.*).<br>• If the data are all contained **within the manuscript and/or Supporting Information files**, enter the following: *All relevant data are within the manuscript and its Supporting Information files.*<br>• If neither of these applies but you are able to provide **details of access elsewhere**, with or without limitations, please do so. For example:<br><br>*Data cannot be shared publicly because of [XXX]. Data are available from the XXX Institutional Data Access / Ethics Committee (contact via XXX) for researchers who meet the criteria for access to confidential data.*<br><br>*The data underlying the results presented in the study are available from (include the name of the third party*	All relevant data are within the manuscript and its Supporting Information files.

and contact information or URL).<br>• This text is appropriate if the data are owned by a third party and authors do not have permission to share the data.<br><br>* typeset	
Additional data availability information:	

Manuscript Click here to access/download;Manuscript;PLOS One_Toba_Final.docx

Coccolithophore assemblage composition during the Greenland Interstadial–Stadial 20 transition and their response to the Youngest Toba Tuff (YTT) supereruption ~74,000 years ago in the northeastern Arabian Sea

Jose Dominick Guballa[1*], Jörg Bollmann[1], Katherine Schmidt[1], Andreas Lückge[2]

[1] Department of Earth Sciences, University of Toronto, Toronto, Ontario, Canada

[2] Bundesanstalt für Geowissenschaften und Rohstoffe (BGR), Hannover, Germany

* Corresponding author

E-mail: doms.guballa@mail.utoronto.ca (JDG)

Author contributions:

JDG - Conceptualization, Formal Analysis, Investigation, Project Administration, Validation, Visualization, Writing – Original Draft Preparation, Writing – Review and Editing

JB - Conceptualization, Funding Acquisition, Methodology, Project Administration, Resources, Supervision, Validation, Writing – Original Draft Preparation, Writing – Review and Editing

KS - Conceptualization, Formal Analysis, Investigation, Methodology, Writing – Review and Editing

AL - Resources, Writing – Review and Editing

Abstract

Here we present a new, 850-year long nearly annually resolved coccolithophore record in core SO130-289KL from the northeastern Arabian Sea that spans the Greenland Interstadial 20–Greenland Stadial 20 transition including the timing of the ~74 ka Youngest Toba Tuff (YTT) supereruption. During the warm interstadial, the coccolithophore assemblage is dominated by *Gephyrocapsa oceanica* (41%) and *Florisphaera profunda* (30%) and changes to small placolith-dominated (*Emiliania huxleyi* + *G. ericsonii*) (52%) in the succeeding cold stadial. However, the oldest samples of the study interval seem to show a stadial-like coccolithophore assemblage. Spectral analysis revealed a bidecadal cycle imprinted in the coccolithophore record during the interstadial that was also independently reported in other terrestrial and marine proxies. Immediately after the YTT eruption, the small placoliths increased by 42% from 5% right below the YTT layer to 47% ~1 mm above the YTT layer, while *G. oceanica* and *Helicosphaera carteri* increased and *F. profunda* decreased within the ~1.15 cm layer representing about 8–19 years.

Subsequently, the coccolithophore assemblage returned to a composition similar to the warm interstadial period before abruptly changing to a stadial composition dominated by small placoliths about 100–150 years after the YTT eruption. Therefore, the YTT eruption had a significant impact on the overall coccolithophore assemblage but appears to have not caused the climate transition from interstadial to stadial conditions, supporting proxy and modeling data. However, the overall mechanism driving the observed changes and cyclicities remains unknown but might be related to rapid atmospheric teleconnections of North Atlantic climate variability to the low latitudes.

Introduction

Anthropogenic greenhouse gas emissions are currently changing climate at an unprecedented rate, impacting ecosystems on a global scale [1]. A natural process capable of such rapid changes are bolide impacts [2] which have driven evolution [3,4] and climate change [5,6] in Earth's history. However, the effects of rapid environmental events such as volcanic supereruptions on climate [7–10], evolution, and the marine ecosystem, especially on marine phytoplankton, are not well constrained.

For example, the most recent supereruption of the Toba volcano, the Youngest Toba Tuff (YTT) eruption ~74 ka [11–13], is the largest supereruption in the Quaternary [14] (Fig 1) but the impact of the eruption on both human evolution [15–17] and global climate [7–10] remain disputed. The YTT eruption was thought to have initiated a global volcanic winter, resulting in the transition from a warm interstadial (Greenland Interstadial 20 or GI-20; [18]) to a cold, stadial climate (Greenland Stadial 20 or GS-20) [9,19]. These climate transitions of the millennial-scale Dansgaard–Oeschger (D–O) oscillations [20] are characterized by abrupt (i.e., decadal-scale) warming and relatively more gradual (i.e., multidecadal to centennial) cooling transitions [18,21], with the latter recently garnering new attention [22] in the context of understanding climate "tipping points" [23].

Fig 1. Extent of the Youngest Toba Tuff (YTT) eruption with respect to the location of core SO130-289KL. The black contour lines represent the projected ashfall thickness in centimeters based on modeling [24]. Dashed lines are estimated extent of the ashfall based on the form of the contours. The red dot approximates the location of core SO130-289KL. Symbols show paleontological and archaeological sites where the impact of the YTT eruption (i.e., ash layer or

signal from proxy record) was reported: ice cores (blue diamonds) [25,26], archaeological sites (orange triangles) [16,27–29], lacustrine sediments (yellow diamonds) [30,31], speleothems (black diamond) [32], and marine sediments (blue dots) [33,34].

The estimated magnitude of global cooling due to SO_4 emissions by the YTT eruption in models ranges considerably, from ~8 °C [10] to ~2–4 °C [7,35] and, more recently, a maximum cooling of up to 1.5 °C to a possible warming scenario of up to 2 °C [8]. However, in contrast to a homogeneous impact, recent modeling studies argue for a globally heterogeneous climatic effect of the YTT eruption [7,35]. For instance, positive excursions in the stable oxygen isotope composition ($\delta^{18}O$) of speleothems [32,36] and sea surface temperature (SST) cooling of 1 °C based on alkenones ($U_k^{37'}$) in the South China Sea [33] suggest a drying and cooling effect, respectively, of the YTT eruption. In contrast, lake records from Africa [30,31] and SST from alkenones in the Arabian Sea [34] indicate minimal to no climatic effects.

More recently, a strengthened Indian winter monsoon after the YTT eruption was proposed to have driven a significant increase in marine primary productivity (PP) in the northeastern Arabian Sea as inferred from the decrease in the relative abundance of the lower photic zone coccolithophore species *Florisphaera profunda* [11]. However, the study only focused on one coccolithophore species for the PP reconstruction, and the impact of the YTT eruption on the overall coccolithophore assemblage has not yet been analyzed even though coccolithophores have been reported to be sensitive to changes in surface water properties [37–40].

Here we present a new dataset on the coccolithophore assemblage composition that spans the GI-20 to GS-20 transition including the timing of the YTT eruption from nearly annually resolved laminated sediments from the northeastern Arabian Sea. This dataset provides new

insights on how geologically instantaneous events such as volcanic eruptions as well as climate tipping points such as D–O transitions have impacted coccolithophores in the past, a prerequisite to assess the response of this important marine phytoplankton group to the current climate crisis.

Materials and methods

Core SO130-289KL (20.2 m long; 23° 07.3' N, 66° 29.8' E) was recovered at 571 m water depth northwest of the Indus River submarine canyon [41] within the center of a stable oxygen minimum zone at the continental slope of the northeastern Arabian Sea [42] (Fig 1). The lack of oxygen in the core location allowed for the preservation of laminated interstadial sediments of nearly annual temporal resolution and the interstadial–stadial transitions are well defined, including the GI-20 to GS-20 transition [43,44]. Furthermore, two ash layers established to be correlated to the YTT eruption enclose ~1.15 cm of laminated sediments as part of a miniature slump fold [44], preserving environmental conditions immediately after the YTT eruption.

A ~1 cm-wide U-channel sampler was used to obtain a subsection between 1870 to 1820 cm core depth (hereafter referred to as cm), which covers the interval before and after the YTT eruption and the transition from Greenland Interstadial 20 to Greenland Stadial 20 [43]. We focused on the interval between 1854.6–1820.4 cm, corresponding to a time period between 74.353 to 73.505 ka with an age estimate of 73.9 ± 0.1 ka (2σ) for the YTT eruption [11]. The 1.15 cm-thick fold between the two YTT layers represents a duration of approximately 8–19 years based on various assumptions on sediment repetition [11].

Coccolithophore analysis

A total of 107 samples were analyzed for coccolithophore assemblage composition and the sampling strategy and sample preparation are described in Guballa et al. [11]. Samples were

analyzed using a Zeiss Axio Imager Z1 transmitted light microscope at 1250x magnification using a PlanApo oil immersion objective (numerical aperture = 1.4) and circularly polarized light [45]. We used standard taxonomic descriptions for coccolithophore species identification [46,47]. At least 300 coccoliths (i.e., isolated calcareous plates of coccolithophores) were counted per sample to calculate the relative abundance for each species and the corresponding 95% confidence intervals were calculated using the Wald interval [48]. We combined the coccolithophore species *Emiliania huxleyi* and *Gephyrocapsa ericsonii* as "small placoliths", as the minute sizes of these species made identification of some specimens on the light microscope difficult. Relative abundances of the species *F. profunda* and *Helicosphaera carteri* are taken from Guballa et al. [11]. All data generated from this study are provided in S1 Table.

Spectral analyses

Spectral analysis was done on the unevenly spaced data set using the "redfit" function [49] in the dplR package version 1.7.2 [50] in R v.4.3.3 [51]. This function avoids linear interpolation that artificially decreases the spectral power of higher frequency components [52]. The analysis was applied to samples prior to the YTT eruption (i.e., 1854.6–1843.3 cm, 74.353–73.886 ka) using coccolithophore species that were consistently present throughout the studied interval. To calculate the spectrum, the default settings were applied except for the number of segments where we only used one due to the relatively few points in the interval (n = 60). Significance testing was performed using the same function by running 2000 Monte Carlo simulations to compute for the 90%, 95%, and 99% red noise false alarm levels.

Results

Core SO130-289KL from the northeastern Arabian Sea is composed of dark-colored, indistinctly to distinctly laminated, carbonate-poor, and organic-rich interstadial sediments from 1854.6–1835 cm core depth and light-colored, homogeneous, bioturbated, carbonate-rich and organic-poor stadial layers from 1835–1820 cm [43,44,53] (Figs 2A and 2B). The YTT eruption is recorded in the core as two discrete, ~1 mm thick ash layers found at 1843.2 and 1842.05 cm enclosing ~1.15 cm of laminated sediments interpreted as a miniature slump fold [44]. Within these sediments, twenty-one (21) coccolithophore species were identified in 107 samples between 1854.6–1820.4 cm (~850-year duration) (S1 Table) and coccolith specimens are generally well-preserved (S1 Fig). Around 90% of the coccolithophore assemblage is comprised of six species: *G. oceanica*, small placoliths belonging to *E. huxleyi* and *G. ericsonii*, *Umbilicosphaera sibogae*, *F. profunda*, and *H. carteri* (Fig 2).

Fig 2. Relative abundance (%) of the main coccolithophore species between 1854.6–1820.4 cm (74.353–73.505 ka) of core SO130-289KL. Data are plotted in relative ages before and after the YTT eruption represented by the lower YTT layer (73.9 ka). (A) Sediment core photo from Deplazes et al. [54] showing clearly visible YTT layers. (B) Sediment L* reflectance [55] showing the lower YTT layer peak (~80% L* reflectance) at 1843.2 cm. (C) *G. oceanica*. (D) small placoliths composed of *E. huxleyi* and *G. ericsonii*. (E) *U. sibogae*. The data for graphs F–H are taken from Guballa et al. [11]: (F) *H. carteri*. (G) *F. profunda*. (H) Annual marine primary productivity (PP) (gC/m^2/yr) based on a single coccolithophore species transfer function [56]. The black dotted lines in B–H show the mean value over the entire study interval. Color bands in C–G demarcate the 95% confidence interval of the data. The gray band shows the miniature slump fold

enclosed by two ash layers from the YTT eruption, with the red dotted line delineating the position of the axial trace. Labels at the far right show informal intervals based on changes in the coccolithophore assemblage composition. Abbreviations: PS = "pseudo-stadial", IS1 = interstadial before the YTT eruption, YTT = Youngest Toba Tuff, IS2 = interstadial after the YTT eruption, S = stadial.

Coccolithophore assemblage composition during Greenland Interstadial 20 (GI-20)

The coccolithophore assemblage from 1854.6–1843.3 cm (~470-year duration) falls within Greenland Interstadial 20 (GI-20). During this time interval, *G. oceanica* is the most common species with an average relative abundance of ~41 ± 5% (Fig 2C). It increased from an average of ~24 ± 5% to an average of ~45 ± 5% around 400 years prior to the eruption and showed high variability until before the YTT eruption. (Fig 2C). Small placoliths (*E. huxleyi* + *G. ericsonii*) are highest (~45 ± 5%) between 470–400 years before the eruption and subsequently remained relatively low (15 ± 4%) until before the eruption (Fig 2D). The species *U. sibogae* varied between ~0.3–19% and peaked around 440–430 years before the eruption (Fig 2E). After a decline between 300–200 years prior to the Toba eruption, *U. sibogae* subsequently returned to previous values except for sharp declines just before the eruption (Fig 2E). Although *H. carteri* is a minor component of the coccolithophore assemblage (<3%), it seems to have a general pattern of occurrence: it was found between 440–300 years before the eruption, undetected from 300–200 years before the eruption, and then reappeared between 200–25 years before the eruption (Fig 2F).

Florisphaera profunda contributed an average of 30 ± 5% to the assemblage, reaching up to 55–60% centered at 250 and 75 years prior to the eruption (Fig 2G).

In general, visual examination of the record during the interstadial shows a shift in the assemblage composition at 1852.8 cm, about 400 years before the YTT eruption (Fig 2). Between 470–400 years before the YTT eruption (1854.6–1852.8 cm), the interstadial assemblage is characterized by the dominance of small placoliths over *G. oceanica* and *F. profunda*. In contrast, the remaining section of the interstadial before the YTT eruption (1852.6–1843.2 cm) is characterized by the dominance of *G. oceanica* and *F. profunda* over the small placoliths.

Coccolithophore assemblage composition immediately after the YTT

In comparison to the interstadial assemblage composition from 1854.6–1843.3 cm, *G. oceanica* increased from a mean value of 41 ± 5% to 60 ± 5% with maximum values of up to 80% within the miniature slump fold (1843.1–1842.1 cm) except for one sample 1 mm above the YTT layer (Fig 2C). In this sample (1843.1 cm) about ~2 years after the YTT eruption, the relative abundance of *G. oceanica* is significantly lower (37 ± 5%) compared to the mean (60 ± 5%) in this interval (Fig 2C). In contrast, small placoliths sharply increased from 5 ± 2% at 1 mm below the YTT layer to 47 ± 6% at 1 mm above the YTT layer, subsequently returning to low values (5–25%) (Fig 2D). *Umbilicosphaera sibogae* reached a maximum of 16 ± 4% in the miniature slump fold (Fig 2E), and *H. carteri* reached up to 7 ± 3% which shows an approximately symmetrical relative abundance pattern within the miniature slump fold (Fig 2F). In contrast, *F. profunda* significantly decreased from an average of 30 ± 5% before the YTT eruption to an average of 8 ± 3% right after the YTT eruption (Fig 2G).

Coccolithophore assemblage composition after the YTT and during Greenland Stadial 20 (GS-20)

The overall coccolithophore assemblage returned to an "interstadial" composition (i.e., 1852.6–1843.2 cm) dominated by *G. oceanica* and *F. profunda* (Fig 2) between 20–100 years after the YTT eruption (1840.9–1836.6 cm) before shifting to a small placolith-dominated assemblage between 100–150 years after the YTT eruption (1836.6–1835 cm). This major shift also coincided with the transition from laminated dark-colored to homogeneous light-colored sediments, characterizing the succeeding cold Greenland 20 Stadial (Figs 2A and 2B). Samples in the stadial interval ~130–370 years after the YTT eruption (1835–1820.4 cm) are characterized by the dominance of small placoliths (45 ± 5%) over *G. oceanica* (30 ± 5%) (Figs 2C and 2D) and low average relative abundance of *U. sibogae* (4 ± 2%; Fig 2E), *H. carteri* (1 ± 1%; Fig. 2F), and *F. profunda* (10 ± 3%; Fig 2G). Variations in the overall coccolithophore assemblage in this interval are clearly driven by changes in *G. oceanica* and the small placoliths (Figs 2C and 2D).

Spectral analysis

Spectral analysis of our data set was done prior to the YTT eruption (i.e., 1854.6–1843.3 cm, 74.353–73.886 ka) since (1) sediments above the ash layer at 1843.2 cm might be disturbed by slumping [44] and may not necessarily represent the correct age and (2) the coccolithophore assemblage right after the YTT eruption (i.e., in the ~1.15 cm interval) might influence the long-term spectral signal and may skew the resulting spectrum. The analysis revealed a 20–22 and 20–21-year spectral band that exceeded the 95% confidence level in *G. oceanica* and small placoliths, respectively (Figs 3A and 3B). In contrast, the power spectrum of *U. sibogae* revealed a significant 28–29-year band (Fig 3C). The power spectrum of *F. profunda* did not show any significant

spectral bands at the 90% confidence level although the 18–19-year band almost reaches the 90% confidence level threshold (Fig 3D).

Fig 3. Spectral analysis of the relative abundance record of the main coccolithophore species. (A) *G. oceanica*. (B) small placoliths consisting of *E. huxleyi* and *G. ericsonii*. (C) *U. sibogae*. (D) *F. profunda*. The black line shows the spectrum for each species. Brown, orange, and red lines represent the 90%, 95%, and 99% confidence limits, respectively. The yellow bands highlight bands exceeding the 90% confidence limit. Yellow bands with asterisks show bands that are very close to the 90% confidence limit.

Discussion

Core SO130-289KL was obtained from a water depth (571 m; [44]) that is significantly above the average lysocline depth (3,300 m; [57]) and we observed that the coccoliths in our samples are mostly well preserved (S1 Fig). Hence, taphonomic effects (i.e., carbonate dissolution) did not cause significant alterations to our data and variations in the coccolith assemblage reflect changes in surface water conditions in the northeastern Arabian Sea. Indeed, this interpretation is supported by combined plankton, sediment trap, and surface sediment analyses in the area which showed that the proportion of the main coccolithophore species in the water column and in the sediments are statistically similar [58].

The coccolithophore assemblage composition in core SO130-289KL compares well with several downcore Holocene and Pleistocene records from the Arabian Sea [59–62] where *G. oceanica*, *F. profunda*, and small placoliths composed of *E. huxleyi* and *G. ericsonii* comprise the major proportion of the coccolithophore assemblage.

However, our record reveals new insights on changes in the coccolithophore assemblage composition immediately after the YTT eruption and in the interstadial–stadial transition (Fig 2). As coccolithophores have been reported to be sensitive to changes in surface water properties [37–40], we assume that our coccolithophore data from the northeastern Arabian Sea reflect the impacts of major environmental perturbations related not only to the YTT eruption, but also to the climatic transition from Greenland Interstadial 20 (GI-20) to Greenland Stadial 20 (GS-20) and potentially to climate oscillations during GI-20.

The present-day climate in the Arabian Sea is primarily driven by differential heating of the Asian Plateau and the Southern Indian Ocean, resulting in seasonally reversing monsoon winds [63]. During boreal summer (June–September), faster heating of the Asian Plateau forms a lower air pressure over the continent relative to the Southern Indian Ocean. This air pressure gradient leads to the warm and humid cross-equatorial jet of the southwest monsoon [64] that drives a clockwise basin-wide surface circulation in the Arabian Sea [65,66] (Figs 4A and 4B). These surface currents and wind stresses cause upwelling along the coasts of Somalia and Oman and promotes primary productivity (PP) in the northwestern Arabian Sea [67,68]. Coastal upwelling also occurs along the coast of Pakistan, on the west side of the northeast–southwest trending Murray Ridge [69]. In general, the upwelling is restricted to the west of the ridge, thus there is relatively low summer PP in the northeastern Arabian Sea east of the Murray Ridge where our core is located [69,70] (Fig 4A).

Fig 4. General seasonal hydrography of the northern Arabian Sea. The red dots in each panel show the location of core SO130-289KL. (A) Summer (June–September) surface ocean circulation [65,66] superimposed on average chlorophyll concentrations (mg/mL^3) between 2005–2017 [71]

as a proxy for primary productivity. (B) Summer average surface wind vectors (m/s, arrows) between 2005–2017 [72] overlain on mixed layer depth (m, contours) between 2005–2017 [73]. (C) As in (A), but for winter (December–February). (D) As in (B) but for winter. Abbreviations: GW = Great Whirl, NMC = Northeast Monsoon Current, RHJ = Ras al Hadd Jet, SC = Somalia Current, SMC = Southwest Monsoon Current, WICC = West India Coastal Current.

During boreal winter (December–February), the air pressure gradients reverse, leading to dry and cold northeasterly winds of the Indian winter monsoon. These winds induce a less prominent counterclockwise basinal surface circulation [65,66] (Fig 4C) and cool sea surface temperatures by ~5 °C from ~29 °C to ~24 °C [74]. The cooling leads to densification and associated convection of surface waters, resulting in a deepening of the mixed layer [75,76] (Fig 4D). The mixed layer deepening entrains nutrients from the subsurface nutricline towards the surface and ultimately increases basin-wide PP in the northern Arabian Sea, including the location of core SO130-289KL (Figs 4C and 4D).

Sediment trap [77,78] and plankton studies [79,80] have revealed that coccolithophores in the Arabian Sea respond to variations in the strength of the monsoons and associated changes in the surface ocean mixed layer depth. For example, the decrease of the lower photic zone species *F. profunda* in core SO130-289KL suggests a deepened mixed layer due to a strengthened Indian winter monsoon that leads to an increase in PP in the northeastern Arabian Sea [11]. Similarly, our data show that the upper photic zone species *G. oceanica* and *H. carteri* reached their highest relative abundance values (80% and 7%, respectively) right after the YTT eruption (Fig 2) concomitant with the proposed deepening of the mixed layer [11]. Subsequently, the coccolithophore assemblage returned to previous “interstadial” compositions dominated by *G.*

oceanica and *F. profunda* that lasted a few decades before shifting towards an assemblage dominated by small placoliths (i.e., *E. huxleyi* and *G. ericsonii*) in the succeeding stadial (Fig 2).

Coccolithophore assemblage changes and interstadial–stadial transitions

The impact of the YTT eruption on the GI-20 to GS-20 transition has been heavily debated on whether it triggered the climate cooling [9,19,81] or only contributed to the cooling [25,26]. In this context, the fact that our data clearly show a significant change in the coccolithophore assemblage immediately after the YTT eruption (Fig 2) strongly suggests that the eruption significantly impacted the marine environment. However, the return of the coccolithophore assemblage to "interstadial" compositions before transitioning to "stadial" compositions supports the view that the YTT eruption was not the trigger for the transition from GI-20 to GS-20 [10,25,26].

During the interstadial (GI-20) to stadial (GS-20) transition, the change in the coccolithophore assemblage composition from *G. oceanica* and *F. profunda* to small placoliths composed of *E. huxleyi* and *G. ericsonii* aligns with many other proxies that recorded this climatic transition (Fig 5). Indeed, the assemblage shift also follows the change in sediment texture from distinctly laminated, dark-colored interstadial sediments to homogeneous, bioturbated, light-colored stadial sediments also reflected by the increasing trend of L* reflectance at the GI-20 to GS-20 transition (Figs 5A and 5C) interpreted as a weakening of the Indian summer monsoon [43,82]. These changes follow within age uncertainties a positive shift in the oxygen isotopic composition ($\delta^{18}O$) of speleothems in China suggesting a weakened Asian summer monsoon [32,83] (Fig 5D), associated with a southward migration of the Intertropical Convergence Zone

(ITCZ) [84–86] and decreased air temperatures recorded in Greenland ice cores [21,87] (Fig 5E). On the other hand, increasing median grain sizes of Chinese loess deposits, a proxy for winter monsoon strength, most likely indicates a strengthened winter monsoon system induced by the interstadial–stadial transition [88–90]. Hence, the alignment of our data with many other proxies (Fig 5) shows the potential of using coccolithophore assemblage changes to trace Dansgaard–Oeschger (D–O) climate transitions in middle to low latitudes.

Fig 5. Comparison of the coccolithophore assemblage to paleo-proxy records between 74.40 to 73.40 ka. (A) small placoliths consisting of *E. huxleyi* and *G. ericsonii*. (B) Annual marine primary productivity (PP) record from core SO130-289KL [11]. (C) L* reflectance record [55]. (D) Stable oxygen isotopic composition ($\delta^{18}O$) of Yangkou YK02 speleothem from southwestern China [32]. (E) North Greenland Ice Core Project (NGRIP) $\delta^{18}O$. The red line is the 20-year mean [21] while the black line is a high-resolution record (4-year resolution) spanning the YTT eruption [91]. In panels A–D, the red lines show trends obtained from running a bootstrap (n = 1000) LOESS smoothing with a 0.1 ka window. The blue rectangle delineates the duration of the miniature slump fold preserved in core SO130-289KL. The violet line indicates the age of the YTT eruption (73.9 ka).

Stadial-like coccolithophore assemblage during an interstadial?

We also detected significant changes in the coccolithophore assemblage that do not seem to be reflected in other proxy records. For instance, within the interstadial prior to the YTT eruption, the coccolithophore assemblage is characterized for about 70 years (470–400 before the YTT) by *G. oceanica* and small placolith abundances very similar to the succeeding “stadial”

assemblage composition (Fig 6). The change in the coccolithophore assemblage aligns well with L* reflectance and primary productivity (Figs 5A–5C) but not with other proxy records (Figs 5D and 5E). We tentatively called this time period a "pseudo-stadial".

Fig 6. Box-whisker plots of the relative abundances (%) of coccolithophore species characteristic for the "pseudo-stadial" (PS; 1854.6–1852.8 cm), the interstadial before the YTT eruption (IS1; 1852.6–1843.3 cm), immediately after the YTT eruption (YTT; 1843.1–1842.1 cm), the interstadial after the YTT eruption (IS2; 1842–1836.6 cm), and the stadial (S; 1835–1820.4 cm). In all cases, n = 10 for PS, n = 50 for IS1, n = 11 for YTT, n = 23 for IS2, and n = 13 for S. (A) *G. oceanica*. (B) small placoliths consisting of *E. huxleyi* and *G. ericsonii*. (C) *U. sibogae*. (D) *H. carteri*. (E) *F. profunda*. Note different y-axis scales. The lower and upper bounds of the boxes represent the first and third quartiles of the data, respectively (i.e., the interquartile range of IQR), and the median is shown as a heavy horizontal line in the box. Notches approximate the 95% confidence interval of the median and are inverted when the 95% confidence interval exceeds the IQR. The red cross and thin grey bar show the mean and 95% confidence interval of the mean, respectively. Tukey style whiskers encompass all data within 1.5 times the IQR. Gray circles represent individual data points while black squares are outliers (i.e., values greater than 1.5 times the IQR). The dotted horizontal line in each panel shows the annual mean relative abundance of each species based on the sediment trap data of Andruleit et al. [77].

It is crucial to note that our record is close to the transition from Marine Isotope Stage (MIS) 5a to MIS 4 ~71 ka [92], characterized by rapid and extensive reorganization of the climate [93] and CO_2 drawdown [94]. Although further independent evidence is required, we speculate

that a transient strengthening of the winter monsoon near the end of a warm interstadial as the climate system started to shift from one mode (i.e., last interglacial period) to another (i.e., last glacial period) [95] might explain the occurrence of the "pseudo-stadial" assemblage. While the $\delta^{18}O$ signature of speleothems do not show similar variability (Fig 5D) [32], loess deposits from northern China show abrupt increases in median grain sizes indicating a strengthened East Asian winter monsoon just before the MIS 5a to MIS 4 transition [90,96]. Therefore, coccolithophore assemblages appear to record not only millennial-scale D–O climate oscillations but also accurately reflect annual to decadal climate variability.

Factors affecting upper photic zone coccolithophore assemblage

The variability of the overall coccolithophore assemblage in the northeastern Arabian Sea is associated with variations in the strength of the monsoons and related changes in the surface ocean mixed layer depth [77,79]. Although mixed layer deepening has been shown to control the ratio of upper photic zone and lower photic zone species in marine sediments [11], there might be additional parameters that contribute to the observed relative abundance changes specifically of upper photic zone species.

Gephyrocapsa oceanica is currently the most abundant coccolithophore species in the Arabian Sea as shown by plankton [79,80,97], sediment trap [77,78], and surface sediment studies [98,99]. Sediment trap data near our core location show maxima in both proportions and coccolith flux of *G. oceanica* during winter [77], concurrent with a deepened mixed layer and increased primary productivity (PP) (Fig 4D) [75,76]. However, *G. oceanica* is composed of at least two distinct morphotypes—possibly different species—each with unique environmental preferences [100–103]. In particular, the *Gephyrocapsa* "Larger" morphotype was reported to occur in nutrient-rich upwelling sites and temperatures ranging from ~18–23 °C [97,100] while the

Gephyrocapsa “Equatorial” morphotype seems to prefer warm water regions (~25–29 °C) [102]. Thus, the assumption that increases in *G. oceanica* reflect solely nutrient-rich conditions [77,79,98,99] might not be entirely correct. For example, we find a clear increase in *G. oceanica* concurrent with a deepened mixed layer right after the YTT eruption (Figs 2C and 6A). However, during the succeeding stadial characterized by a similar water column structure, small placolith species (i.e., *E. huxleyi* and *G. ericsonii*) are dominant (Figs 2D and 6B).

Furthermore, the interpretation of the abundance changes in *H. carteri* in our record is also not straightforward as the ecology of *H. carteri* is not well established. In modern oceans, the distribution of *H. carteri* suggests a preference for warm waters [37,38], turbid conditions [104], and increased nutrient availability [98,105]. This species was also considered opportunistic due to its presence in estuarine environments with elevated nutrient availability [106,107]. The preference of *H. carteri* to increased nutrient availability appears to be corroborated by our data right after the YTT eruption (Figs 2F and 2H), although *H. carteri* was low (<3%) during the succeeding stadial (Fig 6D).

In summary, the observed shifts within the composition of the upper photic zone coccolithophore assemblage may be related to additional factors rather than variations in the mixed layer depth alone. Other processes such as dust input, ash fertilization, light reduction for photosynthesis, regional temperature changes, and evolutionary adaptation may have contributed to the observed changes in the upper photic zone coccolithophore assemblage in core SO130-289KL.

For instance, the short-lived (~2-year duration) and sharp increase of small placolith species comprised of *E. huxleyi* and *G. ericsonii* from 5 ± 2% just below the YTT layer to 47 ± 6% immediately above the YTT layer (Figs 2D and 6B) and their dominance in the succeeding

cold stadial might be related to atmospheric deposition of ash and dust. Carbonate dissolution caused by the ash deposition [108] can be excluded as a main mechanism since this process should lead to an increase of the dissolution-resistant species *F. profunda* [109], contrary to what was previously observed in our record (Figs 2G and 6E) [11]. Thus, it is likely that the increase in small placoliths is a primary response to ash deposition concomitant with the YTT eruption, which might have stimulated the production of phytoplankton as observed for modern-day changes in phytoplankton associated with volcanic ash deposition [110–112]. This mechanism is comparable to modeling studies pointing towards dust deposition as an important factor in stimulating marine primary productivity in the present-day Arabian Sea [113,114] that was also proposed for the Oman upwelling margin during the Last Glacial Maximum [61,115]. Therefore, surface nutrient enrichment from dust deposition might have also commenced in earlier glacials and stadials that are, in general, characterized by increased dust fluxes [53,116,117]. Both ashfall and dust input might have resulted in the growth of small placolith species through micronutrient (e.g., iron) fertilization [110–112] although it is also important to note that culture experiments exploring the effects of volcanic ash on growth rates of *E. huxleyi* showed variable responses [118,119].

Another mechanism that might have contributed to the increase in small placoliths is the change in light availability for photosynthesis. The YTT eruption produced aerosols that must have increased the stratospheric aerosol optical depth (AOD) of the atmosphere [7]. The increased AOD may have reduced light intensities available for photosynthesis and hence may have fostered growth of low light tolerant species. In addition, the ashfall may have increased surface water turbidity that may have further reduced light availability for a short period of time. Small placolith species such as *E. huxleyi* seems to adapt to a wide range of light levels [120] and thus might have gained advantage over other coccolithophore species from reduced light levels immediately after

the YTT eruption. Likewise, the potential increase in dust input during stadials [53,116,117] might have also contributed to increased surface water turbidity and might explain the dominance of small placolith species during the succeeding stadial.

The upper photic zone coccolithophore assemblage could have also been impacted by potential changes in sea surface temperatures (SST). The surface temperature effect of the YTT eruption is still debated, ranging from a cooling of 2–4 °C [7] to a potential warming of 2 °C [8] in models, and negligible (0.3 ± 1.5 °C; [34]) to significant cooling (1 °C; [33]) in alkenone SST proxies. However, the succeeding stadial was characterized by a cooling of ~2.5 °C from 27 °C to 24.5 °C in the northeastern Arabian Sea [34] concomitant with the shift to a small placolith-dominated assemblage in our record (Figs 2D and 6B). Although small placolith species (i.e., *E. huxleyi*) are known to occur over a wide range of temperature and salinity [38,40,121], culture experiments suggest that their growth rates are optimal at ~17–20 °C [122,123]. In contrast, *G. oceanica* is favored by warm SST conditions (> ~22–25 °C) [101,122] and dominates in marginal seas [99] along the West Pacific and in the Red Sea where the temperature ranges from 19–30 °C (mean temperature during plankton sampling = 27.2 °C) [124]. The relatively large drop in SST (~2.5 °C) during the succeeding stadial might have been sufficient to shift *G. oceanica* out of its optimum temperature conditions and be replaced by the small placolith species.

We also cannot simply exclude the effect of evolutionary adaptation on the changes in upper photic zone coccolithophores. In particular, the reported dominance reversal from *G. caribbeanica* to *E. huxleyi* in mid-latitude regions (73 ka) [125] is close to the timing of the YTT eruption (73.9 ka). Indeed, shifts considered equivalent to this dominance reversal (i.e., from *G. ericsonii* to *E. huxleyi*-dominated assemblages) were also identified in the Arabian Sea [60], the tropical Indian Ocean [126,127] and off the coast of South Africa [128,129]. However, our data

show an *E. huxleyi*-dominated small placolith assemblage over the entire study interval (S2 Fig) although the species identification on the light microscope is limited. Our results suggest that (1) the reported dominance reversal might have already occurred and (2) the dominance reversal and the YTT eruption are independent events. Nevertheless, the evolutionary replacement of *G. caribbeanica*/*G. ericsonii* to *E. huxleyi* [125] may partly explain the apparent dominance of *E. huxleyi* over *G. ericsonii* in our record.

Interdecadal cycles in the coccolithophore record during GI-20

Further insights on the potential factors affecting the coccolithophore assemblage in our record may be revealed by spectral analysis. Spectral analysis of the coccolithophore record before the YTT eruption indicates the presence of an interdecadal band (~20–22 and 29 years) (Fig 3). Thus, we also investigated whether other highly temporally resolved proxy records show frequencies at or near these interdecadal bands. Indeed, the L* reflectance [43] and the speleothem $\delta^{18}O$ record [32] also show broadly comparable cyclicities at the 18–19-year band (S3A Fig) and at the 15-year band (S3B Fig), respectively. We also detected significant bands at 29–30 years, 19 years, and 15–16 years in the North Greenland Ice Core Project (NGRIP) $\delta^{18}O$ record (S3C Fig) [91]. The detection of broadly similar bands in continental [32], ice [91], and marine records ([43] and this study) (Fig 3 and S3 Fig) suggests that a global-scale mechanism might have driven the observed cycles although the exact process remains uncertain.

Ice core $\delta^{18}O$ records from Greenland over the past millennium (1303–1961 CE) [130] and over the past 8 ka [131] revealed a 20-year cyclicity associated with variations in North Atlantic SST and the Atlantic Meridional Overturning Circulation (AMOC) that was also supported by climate models [132–134]. It is generally accepted that fluctuations in the strength of the AMOC occur synchronously (within age uncertainties; [135]) with changes in Greenland temperatures

[21] and atmospheric circulation in the high latitude North Atlantic [18]. Therefore, we speculate that rapid atmospheric teleconnections might explain the presence of broadly comparable spectral bands found in continental [32], ice [91], and marine records ([43] and this study) (Fig 3 and S4 Fig).

However, other processes of similar frequencies can also be associated with the observed cyclicity in our record. For example, mechanisms proposed for bidecadal periodicities found in Holocene and Last Glacial Maximum proxy records include solar forcing [136,137] and SST variations in the tropical–extratropical Pacific (Pacific Decadal Oscillation or PDO) [138,139]. Another mechanism is the 18.6-year lunar nodal cycle (near the 20-year periods), as suggested for Late Pleistocene varve thickness data in the Arabian Sea [140] and apparently might also be related to large-scale climatic oscillations such as the El Niño–Southern Oscillation [141] and PDO [142]. On the other hand, the 29-year peak for *U. sibogae* might be related to a 31-year peak found in both gray values and varve thickness from a Holocene marine sediment core in the northeastern Arabian Sea linked to an unknown forcing that likely influenced both terrigenous flux and anoxia/productivity [136].

Conclusions

We report an ~850-year coccolithophore assemblage record in core SO130-289KL from the northeastern Arabian Sea (Fig 1), revealing previously unreported responses of upper photic zone coccolithophore species to the ~73.9 ka Youngest Toba Tuff (YTT) supereruption and the transition from the warm Greenland Interstadial 20 (GI-20) to the cold Greenland Stadial 20 (GS-20). Our results indicate that:

1. Approximately 90% of the coccolithophore assemblage is composed of six species: *G. oceanica*, small placoliths belonging to *E. huxleyi* and *G. ericsonii*, *U. sibogae*, *H. carteri*,

and *F. profunda* (Fig 2). Sediments right after the YTT eruption are characterized by increased *G. oceanica* and *H. carteri*, while small placoliths increased only in the sample immediately above the YTT layer. Sediments from GI-20 are mainly composed of *G. oceanica* (mean ~41%) and *F. profunda* (mean ~30%), while sediments from GS-20 are dominated (mean ~52%) by the small placoliths (Figs 2 and 6).

2. Although the YTT eruption had a significant impact on the coccolithophore assemblage, our results support the view that the eruption was not the trigger for the GI-20 to GS-20 transition. A few decades after the YTT eruption, the coccolithophore assemblage shows a return to an "interstadial" composition dominated by *G. oceanica* and *F. profunda* before shifting towards the small placolith-dominated assemblage of the succeeding stadial (Figs 2 and 6).
3. Our results suggest the potential of coccolithophore assemblage changes as a tracer of Dansgaard–Oeschger (D–O) climate transitions in middle to low latitudes. During the interstadial (GI-20) to stadial (GS-20) transition, the change in the coccolithophore assemblage composition from *G. oceanica* and *F. profunda* to small placoliths composed of *E. huxleyi* and *G. ericsonii* aligns with many other proxies that recorded this climatic transition (Fig 5). The changes in upper photic zone coccolithophore assemblage seem to be related to other factors rather than variations in mixed layer depth alone. Potential mechanisms include atmospheric ash and dust deposition and associated light reduction, cooling of sea surface temperature, and evolutionary adaptation.
4. Spectral analysis of data before the YTT eruption revealed an interdecadal variability (~19–22 years, 29 years) (Fig 3) that compare well with other high temporally resolved proxies in the same time interval, as well as Holocene and Last Glacial Maximum proxy records.

While the exact mechanism remains unknown, a global scale process—such as fast high- to low-latitude atmospheric teleconnections—might potentially transmit such signals to geographically widespread proxy records.

Acknowledgements

We gratefully acknowledge the scientific team and the shipboard captain and crew of the RV SONNE 130 for the acquisition of samples that made this study possible.

References

1. Rosenzweig C, Karoly D, Vicarelli M, Neofotis P, Wu Q, Casassa G, et al. Attributing physical and biological impacts to anthropogenic climate change. Nature. 2008;453: 353–357. doi:10.1038/nature06937

2. Alvarez LW, Alvarez W, Asaro F, Michel HV. Extraterrestrial Cause for the Cretaceous-Tertiary Extinction. Science. 1980;208: 1095–1108. doi:10.1126/science.208.4448.1095

3. Raup DM, Sepkoski JJ. Mass Extinctions in the Marine Fossil Record. Science. 1982;215: 1501–1503. doi:10.1126/science.215.4539.1501

4. Schulte P, Alegret L, Arenillas I, Arz JA, Barton PJ, Bown PR, et al. The Chicxulub Asteroid Impact and Mass Extinction at the Cretaceous-Paleogene Boundary. Science. 2010;327: 1214–1218. doi:10.1126/science.1177265

5. Artemieva N, Morgan J, Expedition 364 Science Party. Quantifying the Release of Climate-Active Gases by Large Meteorite Impacts With a Case Study of Chicxulub. Geophys Res Lett. 2017;44: 10180–10188. doi:10.1002/2017GL074879

6. Zachos JC, Arthur MA, Dean WE. Geochemical evidence for suppression of pelagic marine productivity at the Cretaceous/Tertiary boundary. Nature. 1989;337: 61–64. doi:10.1038/337061a0

7. Black BA, Lamarque J-F, Marsh DR, Schmidt A, Bardeen CG. Global climate disruption and regional climate shelters after the Toba supereruption. Proc Natl Acad Sci. 2021;118: 1–8. doi:10.1073/pnas.2013046118

8. McGraw Z, DallaSanta K, Polvani LM, Tsigaridis K, Orbe C, Bauer SE. Severe Global Cooling After Volcanic Super-Eruptions? The Answer Hinges on Unknown Aerosol Size. J Clim. 2024;37: 1449–1464. doi:10.1175/JCLI-D-23-0116.1

9. Rampino MR, Self S. Climate-Volcanism Feedback and the Toba Eruption of ∼74,000 Years Ago. Quat Res. 1993;40: 269–280. doi:10.1006/qres.1993.1081

10. Robock A, Ammann CM, Oman L, Shindell D, Levis S, Stenchikov G. Did the Toba volcanic eruption of ∼74 ka B.P. produce widespread glaciation? J Geophys Res Atmospheres. 2009;114: 1–9. doi:10.1029/2008JD011652

11. Guballa JD, Bollmann J, Schmidt K, Lückge A. The Toba Eruption 74,000 Years ago Strengthened the Indian Winter Monsoon-Evidence From Coccolithophores. Paleoceanogr Paleoclimatology. 2024;39: e2023PA004823. doi:10.1029/2023PA004823

12. Mark DF, Renne PR, Dymock RC, Smith VC, Simon JI, Morgan LE, et al. High-precision ^{40}Ar/^{39}Ar dating of pleistocene tuffs and temporal anchoring of the Matuyama-Brunhes boundary. Quat Geochronol. 2017;39: 1–23. doi:10.1016/j.quageo.2017.01.002

13. Storey M, Roberts RG, Saidin M. Astronomically calibrated ^{40}Ar/^{39}Ar age for the Toba supereruption and global synchronization of late Quaternary records. Proc Natl Acad Sci. 2012;109: 18684–18688. doi:10.1073/pnas.1208178109

14. Rose WI, Chesner CA. Dispersal of ash in the great Toba eruption, 75 ka. Geology. 1987;15: 913–917. doi:10.1130/0091-7613(1987)15<913:DOAITG>2.0.CO;2

15. Ambrose SH. Late Pleistocene human population bottlenecks, volcanic winter, and differentiation of modern humans. J Hum Evol. 1998;34: 623–651. doi:10.1006/JHEV.1998.0219

16. Kappelman J, Todd LC, Davis CA, Cerling TE, Feseha M, Getahun A, et al. Adaptive foraging behaviours in the Horn of Africa during Toba supereruption. Nature. 2024;628: 365–372. doi:10.1038/s41586-024-07208-3

17. Petraglia M, Korisettar R, Boivin N, Clarkson C, Ditchfield P, Jones S, et al. Middle Paleolithic Assemblages from the Indian Subcontinent Before and After the Toba Super-Eruption. Science. 2007;317: 114–116. doi:10.1126/science.1141564

18. Rasmussen SO, Bigler M, Blockley SP, Blunier T, Buchardt SL, Clausen HB, et al. A stratigraphic framework for abrupt climatic changes during the Last Glacial period based on three synchronized Greenland ice-core records: refining and extending the INTIMATE event stratigraphy. Quat Sci Rev. 2014;106: 14–28. doi:10.1016/j.quascirev.2014.09.007

19. Rampino MR, Self S. Volcanic winter and accelerated glaciation following the Toba super-eruption. Nature. 1992;359: 50–52. doi:10.1038/359050a0

20. Dansgaard W, Johnsen SJ, Clausen HB, Dahl-Jensen D, Gundestrup NS, Hammer CU, et al. Evidence for general instability of past climate from a 250-kyr ice-core record. Nature. 1993;364: 218–220. doi:10.1038/364218a0

21. Andersen KK, Azuma N, Barnola J-M, Bigler M, Biscaye P, Caillon N, et al. High-resolution record of Northern Hemisphere climate extending into the last interglacial period. Nature. 2004;431: 147–151. doi:10.1038/nature02805

22. Mitsui T, Boers N. Statistical precursor signals for Dansgaard–Oeschger cooling transitions. Clim Past. 2024;20: 683–699. doi:10.5194/cp-20-683-2024

23. Intergovernmental Panel on Climate Change (IPCC). Framing, Context, and Methods. Climate Change 2021 – The Physical Science Basis: Working Group I Contribution to the Sixth Assessment Report of the Intergovernmental Panel on Climate Change. Cambridge: Cambridge University Press; 2023. pp. 147–286. doi:10.1017/9781009157896.003

24. Costa A, Smith VC, Macedonio G, Matthews NE. The magnitude and impact of the Youngest Toba Tuff super-eruption. Front Earth Sci. 2014;2: 1–8. doi:10.3389/feart.2014.00016

25. Crick L, Burke A, Hutchison W, Kohno M, Moore KA, Savarino J, et al. New insights into the ~ 74 ka Toba eruption from sulfur isotopes of polar ice cores. Clim Past. 2021;17: 2119–2137. doi:10.5194/cp-17-2119-2021

26. Zielinski GA, Mayewski PA, Meeker LD, Whitlow S, Twickler MS, Taylor K. Potential atmospheric impact of the Toba Mega-Eruption ∼71,000 years ago. Geophys Res Lett. 1996;23: 837–840. doi:10.1029/96GL00706

27. Acharyya SK, Basu PK. Toba Ash on the Indian Subcontinent and Its Implications for Correlation of Late Pleistocene Alluvium. Quat Res. 1993;40: 10–19. doi:10.1006/QRES.1993.1051

28. Clarkson C, Harris C, Li B, Neudorf CM, Roberts RG, Lane CS, et al. Human occupation of northern India spans the Toba super-eruption ~74,000 years ago. Nat Commun. 2020;11: 1–10. doi:10.1038/s41467-020-14668-4

29. Smith EI, Jacobs Z, Johnsen R, Ren M, Fisher EC, Oestmo S, et al. Humans thrived in South Africa through the Toba eruption about 74,000 years ago. Nature. 2018;555: 511–515. doi:10.1038/nature25967

30. Lane CS, Chorn BT, Johnson TC. Ash from the Toba supereruption in Lake Malawi shows no volcanic winter in East Africa at 75 ka. Proc Natl Acad Sci. 2013;110: 8025–8029. doi:10.1073/pnas.1301474110

31. Yost CL, Jackson LJ, Stone JR, Cohen AS. Subdecadal phytolith and charcoal records from Lake Malawi, East Africa imply minimal effects on human evolution from the ∼74 ka Toba supereruption. J Hum Evol. 2018;116: 75–94. doi:10.1016/j.jhevol.2017.11.005

32. Du W, Cheng H, Xu Y, Yang X, Zhang P, Sha L, et al. Timing and structure of the weak Asian Monsoon event about 73,000 years ago. Quat Geochronol. 2019;53: 1–10. doi:10.1016/j.quageo.2019.05.002

33. Huang C-Y, Zhao M, Wang C-C, Wei G. Cooling of the South China Sea by the Toba Eruption and correlation with other climate proxies ~71,000 years ago. Geophys Res Lett. 2001;28: 3915–3918. doi:10.1029/2000GL006113

34. Schulz H, Emeis K-C, Erlenkeuser H, von Rad U, Rolf C. The Toba Volcanic Event and Interstadial/Stadial Climates at the Marine Isotopic Stage 5 to 4 Transition in the Northern Indian Ocean. Quat Res. 2002;57: 22–31. doi:10.1006/qres.2001.2291

35. Timmreck C, Graf H-F, Zanchettin D, Hagemann S, Kleinen T, Krüger K. Climate response to the Toba super-eruption: Regional changes. Quat Int. 2012;258: 30–44. doi:10.1016/j.quaint.2011.10.008

36. Carolin SA, Cobb KM, Adkins JF, Clark B, Conroy JL, Lejau S, et al. Varied Response of Western Pacific Hydrology to Climate Forcings over the Last Glacial Period. Science. 2013;340: 1564–1566. doi:10.1126/science.1233797

37. Brand L. Physiological ecology of marine coccolithophores. In: Winter A, Siesser W, editors. Coccolithophores. Cambridge University Press; 1994. pp. 39–50.

38. McIntyre A, Bé AWH. Modern Coccolithophoridae of the Atlantic Ocean—I. Placoliths and Cyrtoliths. Deep Sea Res Oceanogr Abstr. 1967;14: 561–597. doi:10.1016/0011-7471(67)90065-4

39. Okada H, Honjo S. The distribution of oceanic coccolithophorids in the Pacific. Deep Sea Res Oceanogr Abstr. 1973;20: 355–374. doi:10.1016/0011-7471(73)90059-4

40. Okada H, McIntyre A. Modern Coccolithophores of the Pacific and North Atlantic Oceans. Micropaleontology. 1977;23: 1–55.

41. von Rad U, Doose H. SONNE Cruise SO 130 Cruise Report, MAKRAN II. Hannover: Bundesanstalt für Geowissenschaften und Rohstoffe; 1998 p. 178.

42. von Rad U, Schulz H, Khan AA, Ansari MH, Berner U, Čepek P, et al. Sampling the oxygen minimum zone off Pakistan: glacial-interglacial variations of anoxia and productivity (preliminary results, sonne 90 cruise). Mar Geol. 1995;125: 7–19. doi:10.1016/0025-3227(95)00051-Y

43. Deplazes G, Lückge A, Peterson LC, Timmermann A, Hamann Y, Hughen KA, et al. Links between tropical rainfall and North Atlantic climate during the last glacial period. Nat Geosci. 2013;6: 213–217. doi:10.1038/ngeo1712

44. von Rad U, Burgath K-P, Pervaz M, Schulz H. Discovery of the Toba Ash (c. 70 ka) in a high-resolution core recovering millennial monsoonal variability off Pakistan. In: Clift PD, Kroon D, Gaedicke C, Craig J, editors. The Tectonic and Climatic Evolution of the Arabian Sea Region. Geological Society of London; 2002. pp. 445–461.

45. Bollmann J. Technical Note: Weight approximation of coccoliths using a circular polarizer and interference colour derived retardation estimates – (The CPR Method). Biogeosciences. 2014;11: 1899–1910. doi:10.5194/bg-11-1899-2014

46. Bown PR, editor. Calcareous Nannofossil Biostratigraphy. 1st ed. London: Chapman and Hall/Kluwer Academic Publishers; 1998.

47. Perch-Nielsen K. Cenozoic Calcareous Nannofossils. In: Bolli HM, Sanders JB, Perch-Nielsen K, editors. Plankton Stratigraphy. Cambridge: Cambridge University Press; 1985. pp. 427–554.

48. Sokal RR, Rohlf FJ. Biometry: The Principles and Practice of Statistics in Biological Research. Third ed. New York, N. Y.: W. H. Freeman and Company; 1995.

49. Schulz M, Mudelsee M. REDFIT: estimating red-noise spectra directly from unevenly spaced paleoclimatic time series. Comput Geosci. 2002;28: 421–426. doi:10.1016/S0098-3004(01)00044-9

50. Bunn AG. A dendrochronology program library in R (dplR). Dendrochronologia. 2008;26: 115–124. doi:10.1016/j.dendro.2008.01.002

51. R: A Language and Environment for Statistical Computing. Vienna, Austria: R Foundation for Statistical Computing; 2024. Available: https://www.R-project.org/

52. Schulz M, Stattegger K. Spectrum: spectral analysis of unevenly spaced paleoclimatic time series. Comput Geosci. 1997;23: 929–945. doi:10.1016/S0098-3004(97)00087-3

53. Deplazes G, Lückge A, Stuut J-BW, Pätzold J, Kuhlmann H, Husson D, et al. Weakening and strengthening of the Indian monsoon during Heinrich events and Dansgaard-Oeschger oscillations. Paleoceanography. 2014;29: 99–114. doi:10.1002/2013PA002509

54. Deplazes G, Lückge A, Peterson LC, Timmermann A, Hamann Y, Hughen KA, et al. Documentation of sediment core SO130-289KL. PANGAEA; 2013. doi:10.1594/PANGAEA.816191

55. Deplazes G, Lückge A, Peterson LC, Timmermann A, Hamann Y, Hughen KA, et al. Total reflectance of sediment core SO130-289KL. PANGAEA; 2013. doi:10.1594/PANGAEA.815851

56. Beaufort L, Lancelot Y, Camberlin P, Cayre O, Vincent E, Bassinot F, et al. Insolation Cycles as a Major Control of Equatorial Indian Ocean Primary Production. Science. 1997;278: 1451–1454. doi:10.1126/science.278.5342.1451

57. Cullen JL, Prell WL. Planktonic foraminifera of the northern Indian Ocean: Distribution and preservation in surface sediments. Mar Micropaleontol. 1984;9: 1–52. doi:10.1016/0377-8398(84)90022-7

58. Andruleit HA, Rogalla U, Stäger S. From living communities to fossil assemblages: origin and fate of coccolithophores in the northern Arabian Sea. Micropaleontology. 2004;50: 5–21. doi:10.2113/50.SUPPL_1.5

59. Cabarcos E, Flores JA, Singh AD, Sierro FJ. Monsoonal dynamics and evolution of the primary productivity in the eastern Arabian Sea over the past 30ka. Palaeogeogr Palaeoclimatol Palaeoecol. 2014;411: 249–256. doi:10.1016/j.palaeo.2014.07.006

60. Rogalla U, Andruleit HA. Precessional forcing of coccolithophore assemblages in the northern Arabian Sea: Implications for monsoonal dynamics during the last 200,000 years. Mar Geol. 2005;217: 31–48. doi:10.1016/j.margeo.2005.02.028

61. Zhou X, Duchamp-Alphonse S, Kageyama M, Bassinot F, Doressoundiram F, Kissel C. Variations of Primary Productivity in the Northwestern Arabian Sea During the Last 23,000 Years and Their Paleoclimatological Implications. Paleoceanogr Paleoclimatology. 2022;37: e2022PA004453. doi:10.1029/2022PA004453

62. Zhou X, Duchamp-Alphonse S, Bassinot F, Liu C. Summer and Autumn Insolation as the Pacemaker of Surface Wind and Precipitation Dynamics Over Tropical Indian Ocean During the Holocene: Insights From Paleoproductivity Records and Paleoclimate Simulations. Paleoceanogr Paleoclimatology. 2024;39: e2023PA004786. doi:10.1029/2023PA004786

63. Clemens SC, Prell WL, Murray D, Shimmield G, Weedon G. Forcing mechanisms of the Indian Ocean monsoon. Nature. 1991;353: 720–725. doi:10.1038/353720a0

64. Findlater J. A major low-level air current near the Indian Ocean during the northern summer. Q J R Meteorol Soc. 1969;95: 362–380. doi:10.1002/qj.49709540409

65. Schott FA, Xie S-P, McCreary Jr. JP. Indian Ocean circulation and climate variability. Rev Geophys. 2009;47: 1–46. doi:10.1029/2007RG000245

66. Schott FA, McCreary JP. The monsoon circulation of the Indian Ocean. Prog Oceanogr. 2001;51: 1–123. doi:10.1016/S0079-6611(01)00083-0

67. Barber RT, Marra J, Bidigare RC, Codispoti LA, Halpern D, Johnson Z, et al. Primary productivity and its regulation in the Arabian Sea during 1995. Deep Sea Res Part II Top Stud Oceanogr. 2001;48: 1127–1172. doi:10.1016/S0967-0645(00)00134-X

68. Smith SL, Codispoti LA. Southwest Monsoon of 1979: Chemical and Biological Response of Somali Coastal Waters. Science. 1980;209: 597–600. doi:10.1126/science.209.4456.597

69. Quraishee GS. Circulation in the north Arabian Sea at Murray Ridge during S.W. monsoon. Deep Sea Res Part Oceanogr Res Pap. 1984;31: 651–664. doi:10.1016/0198-0149(84)90033-5

70. Owens NJP, Burkill PH, Mantoura RFC, Woodward EMS, Bellan IE, Aiken J, et al. Size-fractionated primary production and nitrogen assimilation in the northwestern Indian Ocean. Deep Sea Res Part II Top Stud Oceanogr. 1993;40: 697–709. doi:10.1016/0967-0645(93)90053-P

71. Acker JG, Leptoukh G. Online analysis enhances use of NASA Earth science data. Eos Trans Am Geophys Union. 2007;88: 14–17. doi:10.1029/2007EO020003

72. Kalnay E, Kanamitsu M, Kistler R, Collins W, Deaven D, Gandin L, et al. The NCEP/NCAR 40-Year Reanalysis Project. Bull Am Meteorol Soc. 1996;77: 437–472. doi:10.1175/1520-0477(1996)077<0437:TNYRP>2.0.CO;2

73. Boyer TP, Garcia HE, Locarnini RA, Zweng MM, Mishonov AV, Reagan JR, et al. World Ocean Atlas 2018. Mixed Layer Depth. National Centers for Environmental Information (NCEI). 2018. Available: https://www.ncei.noaa.gov/archive/accession/NCEI-WOA18

74. Prakash S, Ramesh R. Is the Arabian Sea getting more productive? Curr Sci. 2007;92: 667–671.

75. Madhupratap M, Kumar SP, Bhattathiri PMA, Kumar MD, Raghukumar S, Nair KKC, et al. Mechanism of the biological response to winter cooling in the northeastern Arabian Sea. Nature. 1996;384: 549–552. doi:10.1038/384549a0

76. Prasanna Kumar S, Ramaiah N, Gauns M, Sarma VVSS, Muraleedharan PM, Raghukumar S, et al. Physical forcing of biological productivity in the Northern Arabian Sea during the Northeast Monsoon. Smith SL, editor. Deep Sea Res Part II Top Stud Oceanogr. 2001;48: 1115–1126. doi:10.1016/S0967-0645(00)00133-8

77. Andruleit HA, von Rad U, Brans A, Ittekkot V. Coccolithophore fluxes from sediment traps in the northeastern Arabian Sea off Pakistan. Mar Micropaleontol. 2000;38: 285–308. doi:10.1016/S0377-8398(00)00007-4

78. Broerse ATC, Brummer G-JA, Hinte JEV. Coccolithophore export production in response to monsoonal upwelling off Somalia (northwestern Indian Ocean). Deep Sea Res Part II Top Stud Oceanogr. 2000;47: 2179–2205. doi:10.1016/S0967-0645(00)00021-7

79. Andruleit HA, Stäger S, Rogalla U, Čepek P. Living coccolithophores in the northern Arabian Sea: ecological tolerances and environmental control. Mar Micropaleontol. 2003;49: 157–181. doi:10.1016/S0377-8398(03)00049-5

80. Kleijne A, Kroon D, Zevenboom W. Phytoplankton and foraminiferal frequencies in northern Indian Ocean and Red Sea surface waters. Neth J Sea Res. 1989;24: 531–539. doi:10.1016/0077-7579(89)90131-2

81. Polyak VJ, Asmerom Y, Lachniet MS. Rapid speleothem $\delta^{13}C$ change in southwestern North America coincident with Greenland stadial 20 and the Toba (Indonesia) supereruption. Geology. 2017;45: 843–846. doi:10.1130/G39149.1

82. Schulz H, von Rad U, Erlenkeuser H. Correlation between Arabian Sea and Greenland climate oscillations of the past 110,000 years. Nature. 1998;393: 54–57. doi:10.1038/31750

83. Cheng H, Edwards RL, Sinha A, Spötl C, Yi L, Chen S, et al. The Asian monsoon over the past 640,000 years and ice age terminations. Nature. 2016;534: 640–646. doi:10.1038/nature18591

84. Broccoli AJ, Dahl KA, Stouffer RJ. Response of the ITCZ to Northern Hemisphere cooling. Geophys Res Lett. 2006;33: 1–4. doi:10.1029/2005GL024546

85. Chiang JCH, Bitz CM. Influence of high latitude ice cover on the marine Intertropical Convergence Zone. Clim Dyn. 2005;25: 477–496. doi:10.1007/s00382-005-0040-5

86. Schneider T, Bischoff T, Haug GH. Migrations and dynamics of the intertropical convergence zone. Nature. 2014;513: 45–53. doi:10.1038/nature13636

87. Kindler P, Guillevic M, Baumgartner M, Schwander J, Landais A, Leuenberger M. Temperature reconstruction from 10 to 120 kyr b2k from the NGRIP ice core. Clim Past. 2014;10: 887–902. doi:10.5194/cp-10-887-2014

88. An Z, Kukla GJ, Porter SC, Xiao J. Magnetic susceptibility evidence of monsoon variation on the Loess Plateau of central China during the last 130,000 years. Quat Res. 1991;36: 29–36. doi:10.1016/0033-5894(91)90015-W

89. Rousseau D-D, Bagniewski W, Sun Y. Detection of abrupt changes in East Asian monsoon from Chinese loess and speleothem records. Glob Planet Change. 2023;227: 104154. doi:10.1016/j.gloplacha.2023.104154

90. Yang S, Ding Z. A 249 kyr stack of eight loess grain size records from northern China documenting millennial-scale climate variability. Geochem Geophys Geosystems. 2014;15: 798–814. doi:10.1002/2013GC005113

91. Svensson A, Bigler M, Blunier T, Clausen HB, Dahl-Jensen D, Fischer H, et al. Direct linking of Greenland and Antarctic ice cores at the Toba eruption (74 ka BP). Clim Past. 2013;9: 749–766. doi:10.5194/cp-9-749-2013

92. Lisiecki LE, Raymo ME. A Pliocene-Pleistocene stack of 57 globally distributed benthic $\delta^{18}O$ records. Paleoceanography. 2005;20: 1–17. doi:10.1029/2004PA001071

93. Shackleton S, Menking JA, Brook E, Buizert C, Dyonisius MN, Petrenko VV, et al. Evolution of mean ocean temperature in Marine Isotope Stage 4. Clim Past. 2021;17: 2273–2289. doi:10.5194/cp-17-2273-2021

94. Bereiter B, Lüthi D, Siegrist M, Schüpbach S, Stocker TF, Fischer H. Mode change of millennial CO_2 variability during the last glacial cycle associated with a bipolar marine carbon seesaw. Proc Natl Acad Sci. 2012;109: 9755–9760. doi:10.1073/pnas.1204069109

95. Adkins JF. The role of deep ocean circulation in setting glacial climates. Paleoceanography. 2013;28: 539–561. doi:10.1002/palo.20046

96. Xiong S, Ding Z, Liu T, Zhang J. East Asian monsoon instability at the stage 5a/4 transition. Boreas. 2002;31: 126–132. doi:10.1111/j.1502-3885.2002.tb01061.x

97. Schiebel R, Zeltner A, Treppke UF, Waniek JJ, Bollmann J, Rixen T, et al. Distribution of diatoms, coccolithophores and planktic foraminifers along a trophic gradient during SW monsoon in the Arabian Sea. Mar Micropaleontol. 2004;51: 345–371. doi:10.1016/j.marmicro.2004.02.001

98. Andruleit HA, Rogalla U. Coccolithophores in surface sediments of the Arabian Sea in relation to environmental gradients in surface waters. Mar Geol. 2002;186: 505–526. doi:10.1016/S0025-3227(02)00312-2

99. Houghton SD, Guptha MVS. Monsoonal and fertility controls on Recent marginal sea and continental shelf coccolith assemblages from the western Pacific and northern Indian oceans. Mar Geol. 1991;97: 251–259. doi:10.1016/0025-3227(91)90119-O

100. Bollmann J. Morphology and biogeography of *Gephyrocapsa* coccoliths in Holocene sediments. Mar Micropaleontol. 1997;29: 319–350. doi:10.1016/S0377-8398(96)00028-X

101. Bollmann J, Klaas C, Brand LE. Morphological and Physiological Characteristics of *Gephyrocapsa oceanica* var. *typica* Kamptner 1943 in Culture Experiments: Evidence for Genotypic Variability. Protist. 2010;161: 78–90. doi:10.1016/j.protis.2009.08.002

102. Bollmann J, Klaas C. Morphological Variation of *Gephyrocapsa oceanica* Kamptner 1943 in Plankton Samples: Implications for Ecologic and Taxonomic Interpretations. Protist. 2008;159: 369–381. doi:10.1016/j.protis.2008.02.001

103. Henderiks J, Bollmann J. The *Gephyrocapsa* sea surface palaeothermometer put to the test: comparison with alkenone and foraminifera proxies off NW Africa. Mar Micropaleontol. 2004;50: 161–184. doi:10.1016/S0377-8398(03)00070-7

104. Ziveri P, Thunell RC, Rio D. Export production of coccolithophores in an upwelling region: Results from San Pedro Basin, Southern California Borderlands. Mar Micropaleontol. 1995;24: 335–358. doi:10.1016/0377-8398(94)00017-H

105. Ziveri P, Baumann K-H, Böckel B, Bollmann J, Young JR. Biogeography of selected Holocene coccoliths in the Atlantic Ocean. In: Thierstein HR, Young JR, editors. Coccolithophores: From Molecular Processes to Global Impact. Berlin, Heidelberg: Springer Berlin Heidelberg; 2004. pp. 403–428. Available: https://doi.org/10.1007/978-3-662-06278-4_15

106. Dimiza MD, Triantaphyllou MV, Malinverno E. New evidence for the ecology of *Helicosphaera carteri* in polluted coastal environments. In: Young JR, Gallagher LT, editors. Coccolithophores 2014 workshop volume. Heraklion, Crete, Greece: Journal of Nannoplankton Research; 2014. pp. 37–43. doi:10.58998/jnr2081

107. Guerreiro C, Cachão M. Calcareous nannoplankton as a tracer of the marine influence on the NW coast of Portugal over the last 14 000 years. J Nannoplankton Res. 2005;27: 159–172. doi:10.58998/jnr2107

108. Cobianchi M, Mancin N, Lupi C, Bordiga M, Bostock HC. Effects of oceanic circulation and volcanic ash-fall on calcite dissolution in bathyal sediments from the SW Pacific Ocean over the last 550 ka. Palaeogeogr Palaeoclimatol Palaeoecol. 2015;429: 72–82. doi:10.1016/j.palaeo.2015.03.045

109. Thomson J, Crudeli D, de Lange GJ, Slomp CP, Erba E, Corselli C, et al. *Florisphaera profunda* and the origin and diagenesis of carbonate phases in eastern Mediterranean sapropel units. Paleoceanography. 2004;19. doi:10.1029/2003PA000976

110. Achterberg EP, Moore CM, Henson SA, Steigenberger S, Stohl A, Eckhardt S, et al. Natural iron fertilization by the Eyjafjallajökull volcanic eruption. Geophys Res Lett. 2013;40: 921–926. doi:10.1002/grl.50221

111. Langmann B, Zakšek K, Hort M, Duggen S. Volcanic ash as fertiliser for the surface ocean. Atmospheric Chem Phys. 2010;10: 3891–3899. doi:10.5194/acp-10-3891-2010

112. Spirakis CS. Iron fertilization with volcanic ash? Eos Trans Am Geophys Union. 1991;72: 525. doi:10.1029/90EO00370

113. Banerjee P, Prasanna Kumar S. Dust-induced episodic phytoplankton blooms in the Arabian Sea during winter monsoon. J Geophys Res Oceans. 2014;119: 7123–7138. doi:10.1002/2014JC010304

114. Guieu C, Al Azhar M, Aumont O, Mahowald N, Levy M, Ethé C, et al. Major Impact of Dust Deposition on the Productivity of the Arabian Sea. Geophys Res Lett. 2019;46: 6736–6744. doi:10.1029/2019GL082770

115. Tamburini F, Föllmi KB, Adatte T, Bernasconi SM, Steinmann P. Sedimentary phosphorus record from the Oman margin: New evidence of high productivity during glacial periods. Paleoceanography. 2003;18. doi:10.1029/2000PA000616

116. Clemens SC, Prell WL. Late Pleistocene variability of Arabian Sea summer monsoon winds and continental aridity: Eolian records from the lithogenic component of deep-sea sediments. Paleoceanography. 1990;5: 109–145. doi:10.1029/PA005i002p00109

117. Pourmand A, Marcantonio F, Schulz H. Variations in productivity and eolian fluxes in the northeastern Arabian Sea during the past 110 ka. Earth Planet Sci Lett. 2004;221: 39–54. doi:10.1016/S0012-821X(04)00109-8

118. Faucher G, Hoffmann L, Bach LT, Bottini C, Erba E, Riebesell U. Impact of trace metal concentrations on coccolithophore growth and morphology: laboratory simulations of Cretaceous stress. Biogeosciences. 2017;14: 3603–3613. doi:10.5194/bg-14-3603-2017

119. Hoffmann LJ, Breitbarth E, Ardelan MV, Duggen S, Olgun N, Hassellöv M, et al. Influence of trace metal release from volcanic ash on growth of *Thalassiosira pseudonana* and *Emiliania huxleyi*. Mar Chem. 2012;132–133: 28–33. doi:10.1016/j.marchem.2012.02.003

120. Nanninga HJ, Tyrrell T. Importance of light for the formation of algal blooms by *Emiliania huxleyi*. Mar Ecol Prog Ser. 1996;136: 195–203. doi:10.3354/meps136195

121. Fielding SR. *Emiliania huxleyi* specific growth rate dependence on temperature. Limnol Oceanogr. 2013;58: 663–666. doi:10.4319/lo.2013.58.2.0663

122. Buitenhuis ET, Pangerc T, Franklin DJ, Le Quéré C, Malin G. Growth rates of six coccolithophorid strains as a function of temperature. Limnol Oceanogr. 2008;53: 1181–1185. doi:10.4319/lo.2008.53.3.1181

123. Langer G, Nehrke G, Probert I, Ly J, Ziveri P. Strain-specific responses of *Emiliania huxleyi* to changing seawater carbonate chemistry. Biogeosciences. 2009;6: 2637–2646. doi:10.5194/bg-6-2637-2009

124. Okada H, Honjo S. Distribution of coccolithophores in marginal seas along the western Pacific Ocean and in the Red Sea. Mar Biol. 1975;31: 271–285. doi:10.1007/BF00387154

125. Thierstein HR, Geitzenauer KR, Molfino B, Shackleton NJ. Global synchroneity of late Quaternary coccolith datum levels: Validation by oxygen isotopes. Geology. 1977;5: 400–404. doi:10.1130/0091-7613(1977)5<400:GSOLQC>2.0.CO;2

126. Andruleit HA, Lückge A, Wiedicke M, Stäger S. Late Quaternary development of the Java upwelling system (eastern Indian Ocean) as revealed by coccolithophores. Mar Micropaleontol. 2008;69: 3–15. doi:10.1016/j.marmicro.2007.11.005

127. Tangunan D, Baumann K-H, Pätzold J, Henrich R, Kucera M, De Pol-Holz R, et al. Insolation forcing of coccolithophore productivity in the western tropical Indian Ocean over the last two glacial-interglacial cycles. Paleoceanography. 2017;32: 692–709. doi:10.1002/2017PA003102

128. Flores J-A, Gersonde R, Sierro FJ. Pleistocene fluctuations in the Agulhas Current Retroflection based on the calcareous plankton record. Mar Micropaleontol. 1999;37: 1–22. doi:10.1016/S0377-8398(99)00012-2

129. Tangunan D, Baumann K-H, Fink C. Variations in coccolithophore productivity off South Africa over the last 500 kyr. Mar Micropaleontol. 2020;160: 101909. doi:10.1016/j.marmicro.2020.101909

130. Chylek P, Folland CK, Dijkstra HA, Lesins G, Dubey MK. Ice-core data evidence for a prominent near 20 year time-scale of the Atlantic Multidecadal Oscillation. Geophys Res Lett. 2011;38. doi:10.1029/2011GL047501

131. Hughes AG, Jones TR, Vinther BM, Gkinis V, Stevens CM, Morris V, et al. High-frequency climate variability in the Holocene from a coastal-dome ice core in east-central Greenland. Clim Past. 2020;16: 1369–1386. doi:10.5194/cp-16-1369-2020

132. Arzel O, England MH, de Verdière AC, Huck T. Abrupt millennial variability and interdecadal-interstadial oscillations in a global coupled model: sensitivity to the background climate state. Clim Dyn. 2012;39: 259–275. doi:10.1007/s00382-011-1117-y

133. Frankcombe LM, von der Heydt A, Dijkstra HA. North Atlantic Multidecadal Climate Variability: An Investigation of Dominant Time Scales and Processes. J Clim. 2010;23: 3626–3638. doi:10.1175/2010JCLI3471.1

134. Ortega P, Mignot J, Swingedouw D, Sévellec F, Guilyardi E. Reconciling two alternative mechanisms behind bi-decadal variability in the North Atlantic. Prog Oceanogr. 2015;137: 237–249. doi:10.1016/j.pocean.2015.06.009

135. Fohlmeister J, Sekhon N, Columbu A, Vettoretti G, Weitzel N, Rehfeld K, et al. Global reorganization of atmospheric circulation during Dansgaard–Oeschger cycles. Proc Natl Acad Sci. 2023;120: e2302283120. doi:10.1073/pnas.2302283120

136. von Rad U, Schaaf M, Michels KH, Schulz H, Berger WH, Sirocko F. A 5000-yr Record of Climate Change in Varved Sediments from the Oxygen Minimum Zone off Pakistan, Northeastern Arabian Sea. Quat Res. 1999;51: 39–53. doi:10.1006/qres.1998.2016

137. Sinha A, Cannariato KG, Stott LD, Li H-C, You C-F, Cheng H, et al. Variability of Southwest Indian summer monsoon precipitation during the Bølling-Ållerød. Geology. 2005;33: 813–816. doi:10.1130/G21498.1

138. Biondi F, Gershunov A, Cayan DR. North Pacific Decadal Climate Variability since 1661. J Clim. 2001;14: 5–10. doi:10.1175/1520-0442(2001)014<0005:NPDCVS>2.0.CO;2

139. D’Arrigo R, Villalba R, Wiles G. Tree-ring estimates of Pacific decadal climate variability. Clim Dyn. 2001;18: 219–224. doi:10.1007/s003820100177

140. Berger WH, von Rad U. Decadal to millennial cyclicity in varves and turbidites from the Arabian Sea: hypothesis of tidal origin. Glob Planet Change. 2002;34: 313–325. doi:10.1016/S0921-8181(02)00122-4

141. Yasuda I. Impact of the astronomical lunar 18.6-yr tidal cycle on El-Niño and Southern Oscillation. Sci Rep. 2018;8: 15206. doi:10.1038/s41598-018-33526-4

142. Cook ER, Meko DM, Stockton CW. A New Assessment of Possible Solar and Lunar Forcing of the Bidecadal Drought Rhythm in the Western United States. J Clim. 1997;10: 1343–1356. doi:10.1175/1520-0442(1997)010<1343:ANAOPS>2.0.CO;2

Supporting information

S1 Fig. Scanning electron microscope (SEM) images of common coccolithophore species identified in the study interval in core SO130-289KL. In all panels, the scale bar is 5 μm. (A) *Calcidiscus leptoporus*, 1842.7 cm core depth. (B) *Emiliania huxleyi*, 1842.7 cm core depth. (C) *Gephyrocapsa ericsonii*, 1843.1 cm core depth. (D) *G. oceanica*, 1843.7 cm core depth. (E) *Florisphaera profunda* var. *elongata*, 1843.1 cm core depth. (F) *F. profunda* var. *profunda*, 1840.7 cm core depth. (G) *Helicosphaera carteri*, 1843.1 cm core depth. (H) *Umbilicosphaera sibogae*, 1842.2 cm core depth.

S2 Fig. Relative abundance (%) of the small placolith components in the studied interval of core SO130-289KL. (A) Sediment core scan from Deplazes et al. (2013a) showing clearly visible YTT layers. (B) *E. huxleyi*. (C) *G. ericsonii*. Color bands for each species demarcate the 95% confidence interval for the relative abundances. The gray band shows the miniature slump fold enclosed by two ash layers from the YTT eruption, with the axial trace delineated by the red dotted line.

S3 Fig. Spectral analysis of various marine, terrestrial, and ice records within the same time interval as the coccolithophore record. Note different axis limits. (A) L* reflectance [43]. (B) The YK02 speleothem stable oxygen isotope ($\delta^{18}O$) record from southwest China [32]. (C) North Greenland Ice Core Project (NGRIP) $\delta^{18}O$ record (Svensson et al., 2013). The black line shows the spectrum for each species. Brown, orange, and red lines represent the 90%, 95%, and 99% confidence limits, respectively. The yellow bands highlight bands exceeding the 90% confidence limit.

S1 Table. Coccolithophore relative abundance data from core SO130-289KL from 1854.6 cm to 1820.4 cm.

Figure 1

Click here to access/download;Figure;Fig1.tif

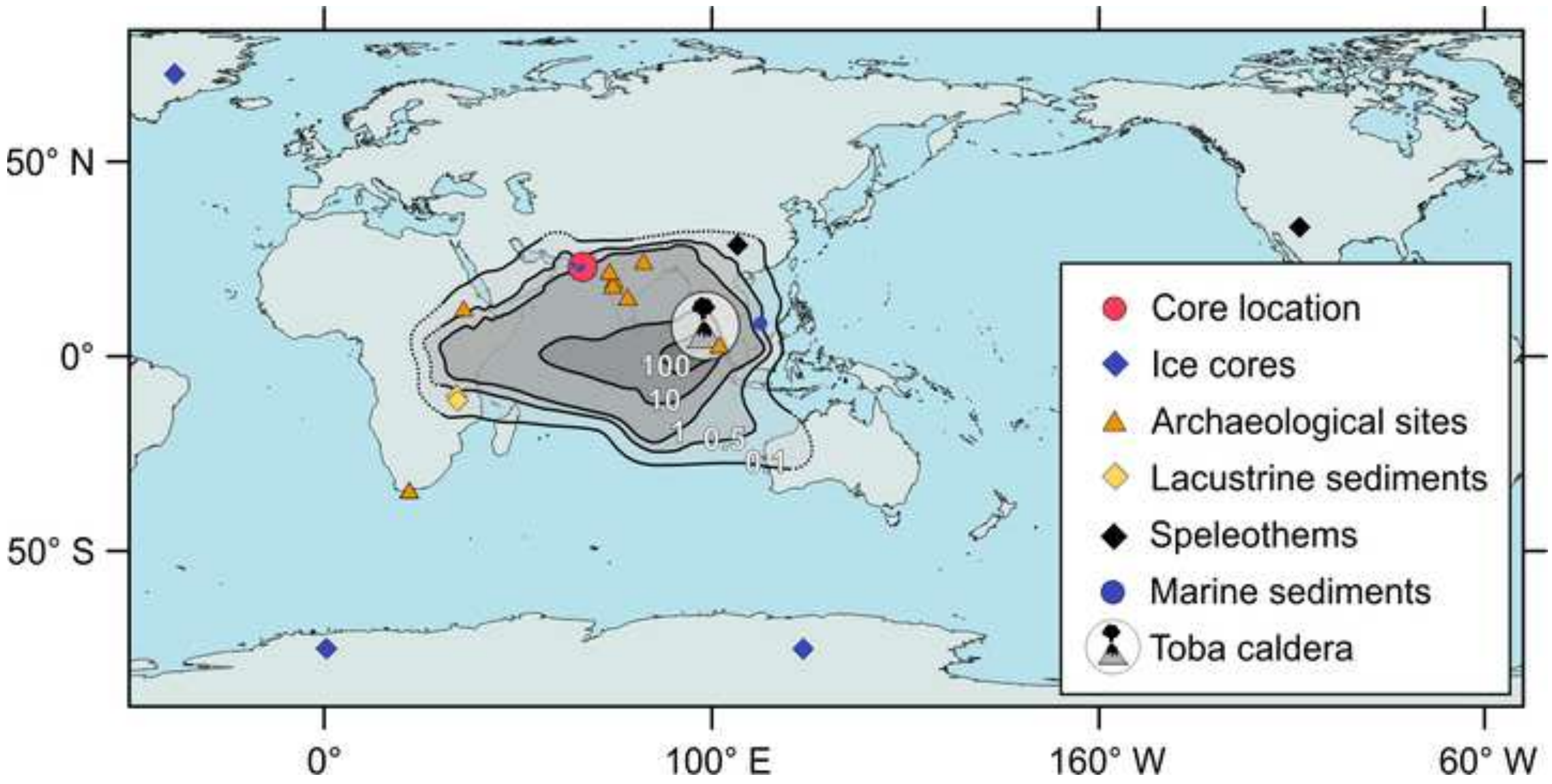

Figure 2

Click here to access/download;Figure;Fig2.tif

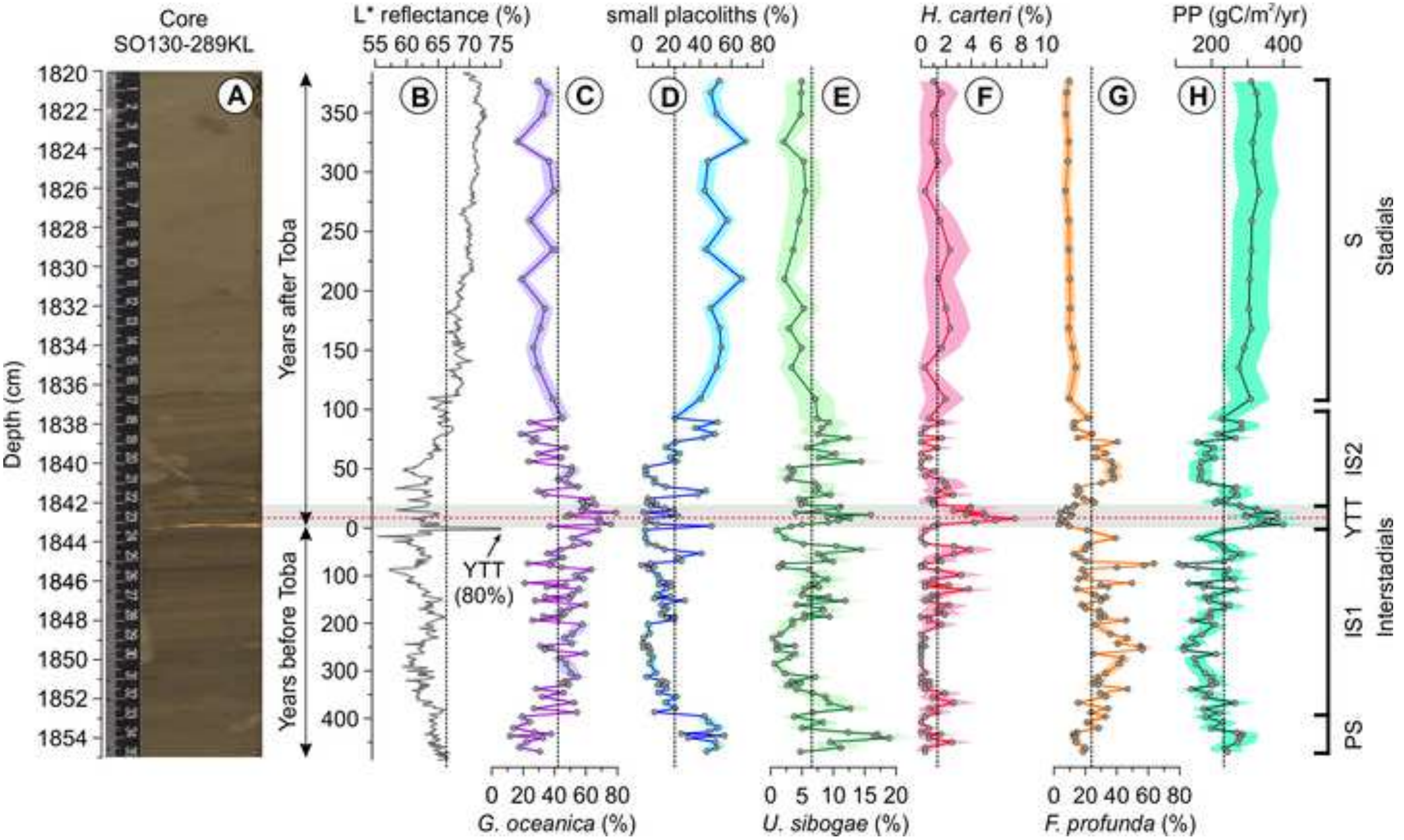

Figure 3

Click here to access/download;Figure;Fig3.tif

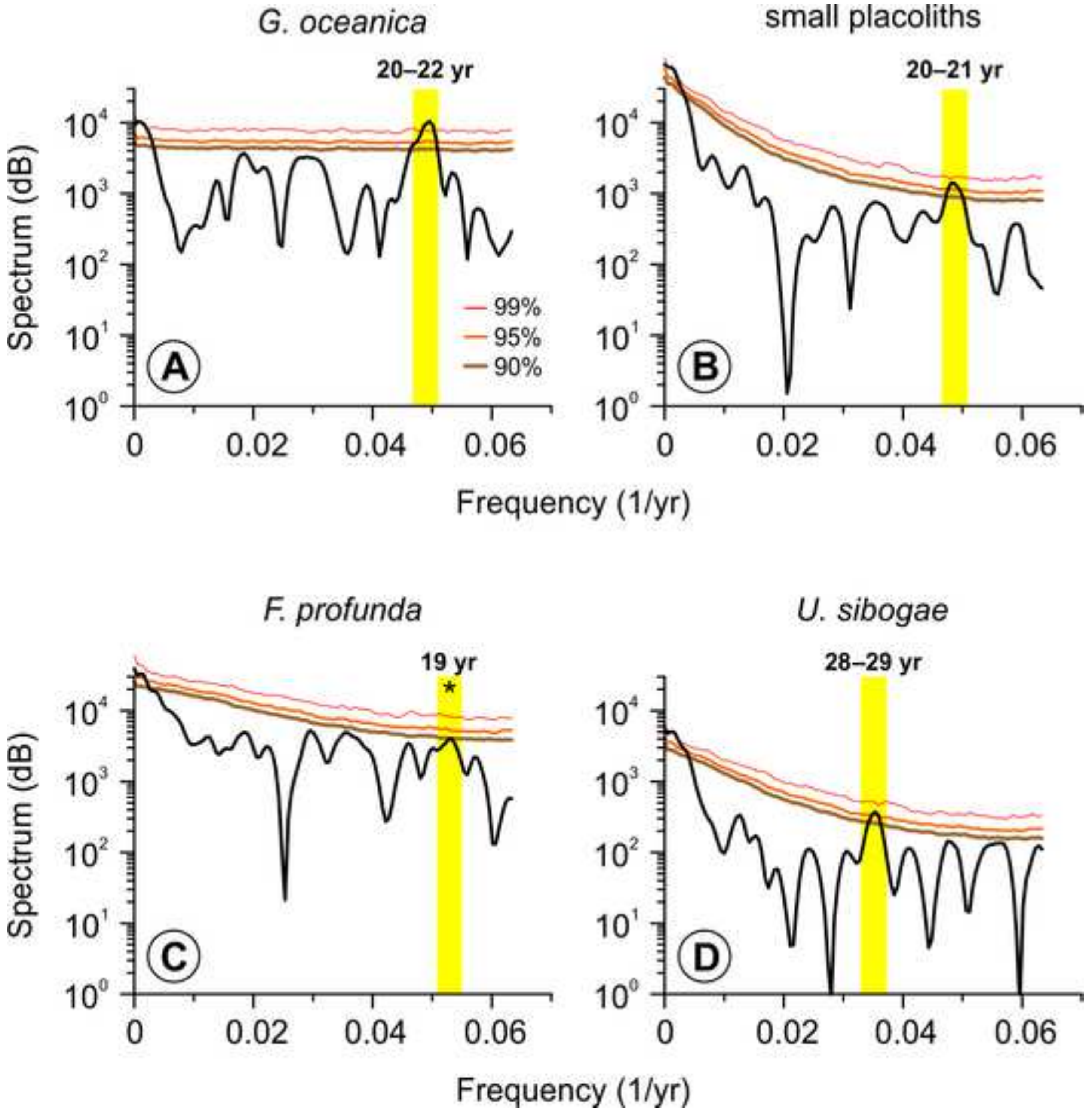

Figure 4

Click here to access/download;Figure;Fig4.tif

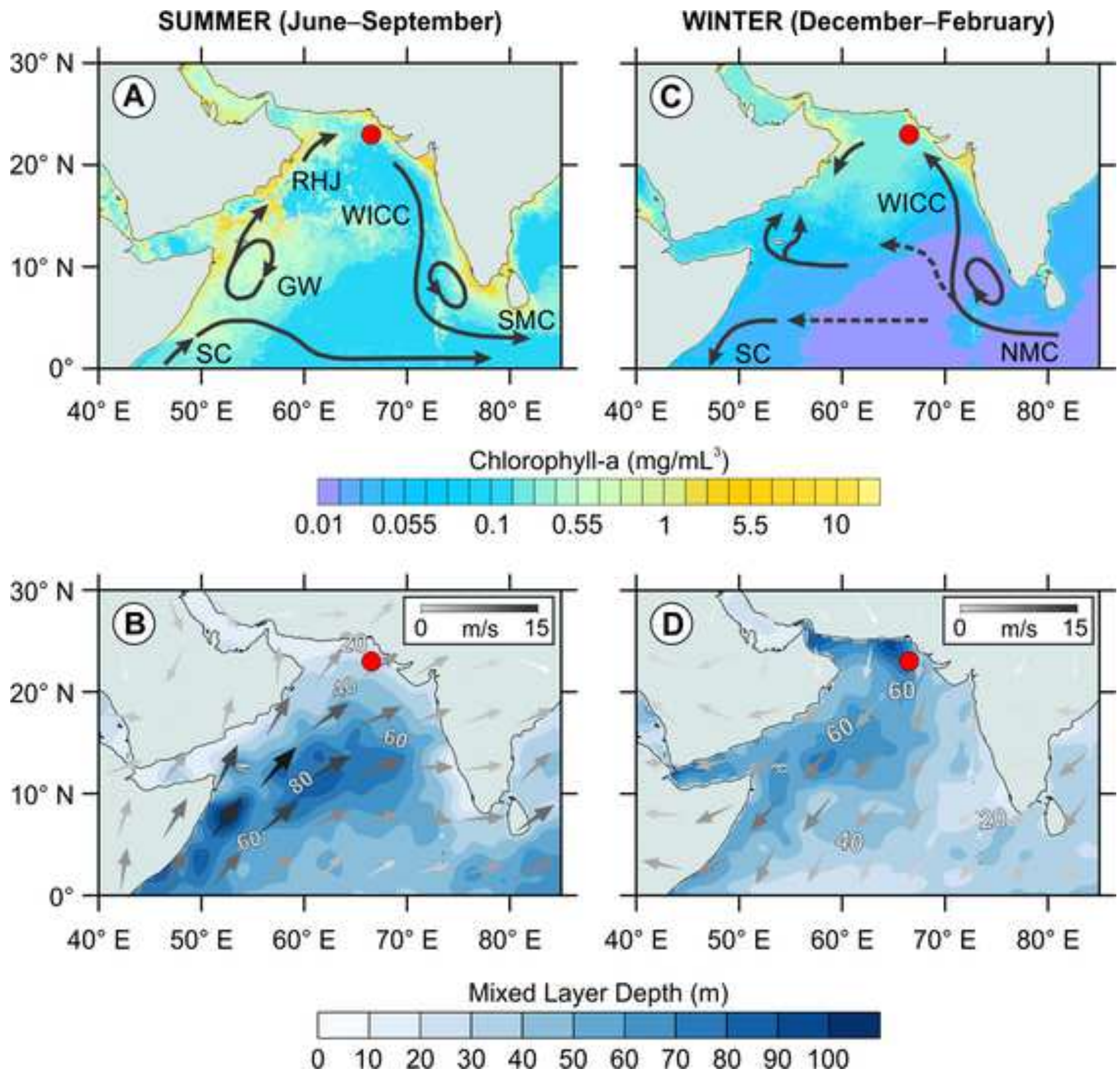

 Click here to access/download;Figure;Fig5.tif

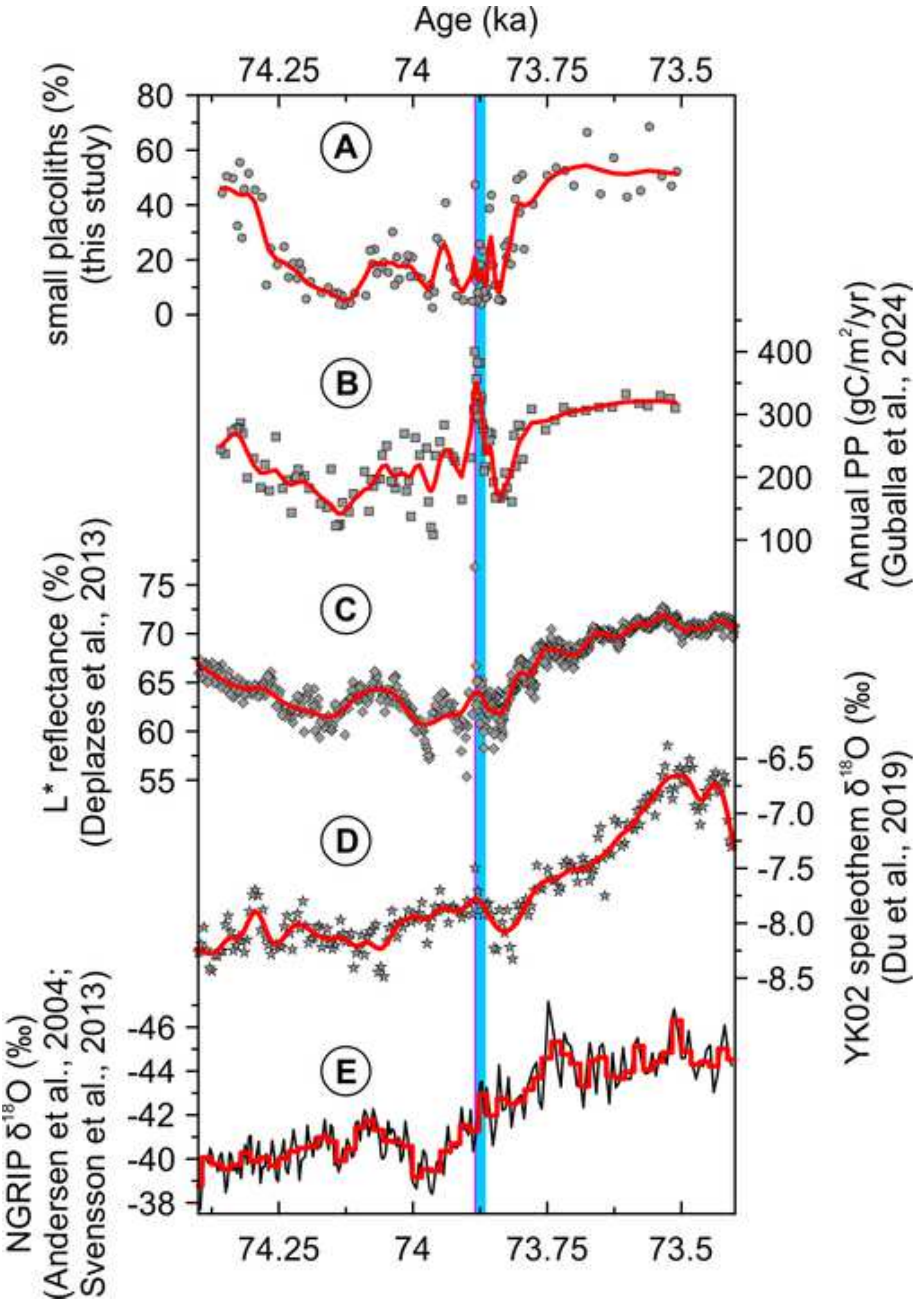

Figure 6

Click here to access/download;Figure;Fig6.tif

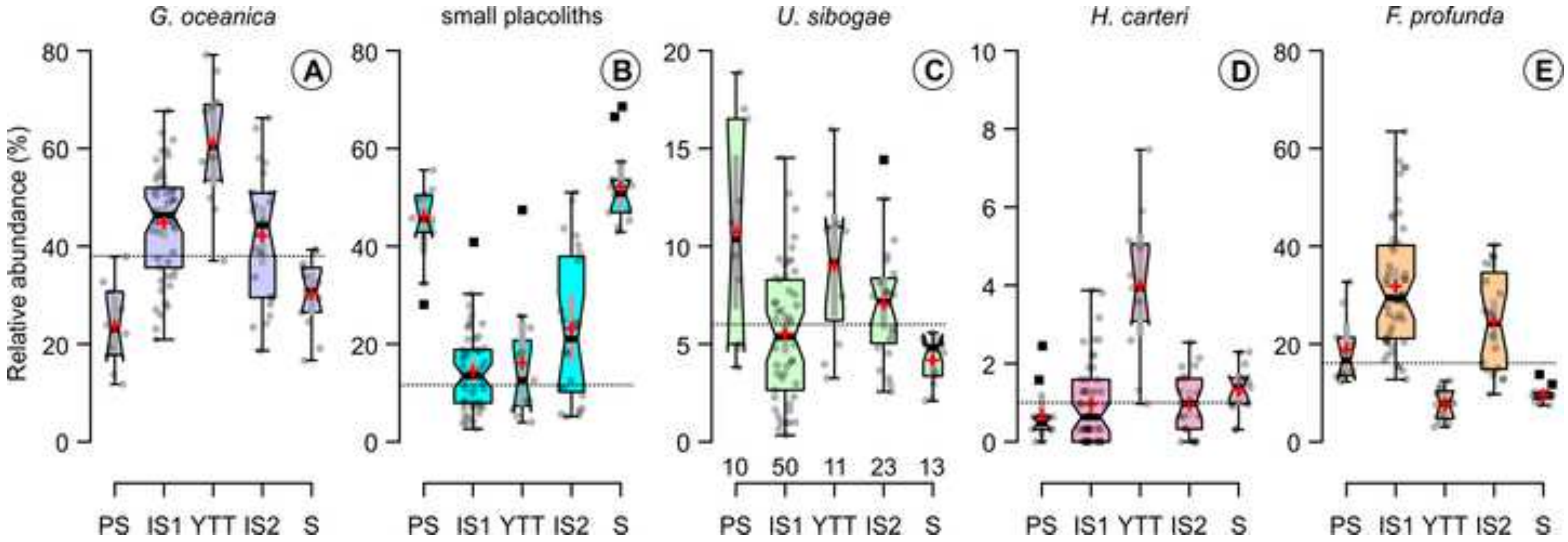

Supporting Information Figure 1

Click here to access/download

Supporting Information

S1_Fig.tif

Supporting Information Figure 2

Click here to access/download

Supporting Information

S2_Fig.tif

Supporting Information Figure 3

Click here to access/download
Supporting Information
S3_Fig.tif

Supporting Information Table 1

Click here to access/download
Supporting Information
S1 Table.xlsx
