# Supplementary material for: Coccolithophore assemblage composition during the Greenland Interstadial–Stadial 20 transition and their response to the Youngest Toba Tuff (YTT) supereruption ∼74,000 years ago in the northeastern Arabian Sea
Source: PLoS One. 2024 Sep 25;19(9):e0310041. doi: 10.1371/journal.pone.0310041 (PMC11423970; doi:10.1371/journal.pone.0310041)
Supplement: Attachment — Submitted filename: Response to Reviewers.docx [file pone.0310041.s011.docx]

We appreciate the helpful comments by the editor and two reviewers that substantially helped in improving our paper. We have addressed the comments/concerns in detail below.

In all cases, the reviewer comments are in black font, while our responses are in blue. Reviewer comments are numbered, and this numbering restarts for each reviewer. Revisions made on the manuscript lines are underlined.

Editor:

Both reviewers much appreciated your work, and i share this feeling. However, there is something to be improved, before publication. Please pay special attention to comments by Reviewer#2, who disputes spectral analysis results. Moreover, in my opinion U. sibogae paleoenvironmental indication is overlooked. Following best literature (you fully cite), U. sibogae shows a positive response, both recorded in relative and absolute abundances, to the onset of the NE monsoon (Andruleit et al., 2000). The cluster analysis carried out on surface sediments associates this species to G. oceanica, within a group characterized by environmental conditions with high nutrient availability (Andruleit and Rogalla, 2002). Thus, this would match your reconstruction, especially at YTT.

**Reply:** We thank the editor for the positive comments on our manuscript. In this document we address the minor concerns of Reviewer #1 and the major concerns of Reviewer #2 especially on spectral analysis.

We refrained from the interpretation of *U. sibogae* since our data are solely based on relative abundances and thus their ecological interpretation is limited by the “unit-sum constraint” (Aitchison, 1994) where the loss of one species is automatically the gain of another species as the sum of all the species must always be a constant (in this case, 100%). This limitation becomes very apparent in our data set. Although the increase in the relative abundance of *U. sibogae* match interpretations of increased primary productivity immediately after the Youngest Toba Tuff (YTT) eruption, the decrease in the relative abundance of *U. sibogae* in the succeeding stadial seems contradictory to the interpretation of a strengthened winter monsoon during this time.

Reviewer #1 Evaluations (Mário Cachão):

1. I have no major criticisms of your manuscript, which I generally find to be very good. I have made a few comments on the attached copy.

**Reply:** We thank Dr. Cachão for the positive and constructive comments on our manuscript. We have also included the comments from the attached copy in this letter.

2. Line 120: Additional notes should be added to the treatment of the less abundant (“Other coccolithophore species”) present on the provided data matrix.

For instance, did you grouped all the other identified Umbilicosphaera species within the U.sibogae or not?

**Reply:** Thank you for the comment. We added the lines below for clarification:

“The different species of *Umbilicosphaera* (i.e., *U. sibogae*, *U. foliosa*) were lumped as *U. sibogae*.”

3. Line 166: I also see similar degree of variability soon after YTT. For layers above 100 years after YTT sample resolution is much lower and so it may give the appearance that the values are more stable.

**Reply:** Reviewer #1 referred to the lines below in red for this comment:

“During this time interval, *G. oceanica* is the most common species with an average relative abundance of ~41 ± 5% (Fig 2C). It increased from an average of ~24 ± 5% to an average of ~45 ± 5% around 400 years prior to the eruption and showed high variability until before the YTT eruption.”

We agree that sample aliasing affected the degree of variability observed in our record. However, we note that samples ~100 years after the YTT are found within the succeeding stadial, characterized by light-colored bioturbated sediments which may have also contributed to the signal smoothing. Furthermore, we do not state in our manuscript that the variability of *G. oceanica* after the YTT was lower than before the YTT. We simply state that the coccolithophore assemblage after the YTT was comparable to that before the YTT and we do not make any statements about changes in variability.

5. Line 394: YES! This (i.e., dust input, ash fertilization) should be stressed in this paper.

Coinciding with YTT you have maximum percentages of G.oceanica, increase in H.carteri (previous study) in accordance with our findings off Iberia. An increase also on the oligotrophic “stratified water loving” U.sibogae means that this cannot be related to an increase on mixing upwelling waters.

**Reply:** We believe we have highlighted these processes in lines 398–426 of our manuscript.

The statement above clearly shows the limitations of % interpretations caused by “unit-sum constraint” (Aitchison, 1994) since the increase in *U. sibogae* might be driven by the loss of other species (i.e., *F. profunda*). It is also very difficult to place an exact ecological preference for *U. sibogae*, as sediment traps in the northeastern Arabian Sea show that it might be responding positively to high nutrient conditions during the winter monsoon (Andruleit et al., 2000) while in the northwestern Arabian Sea, *U. sibogae* appears to not thrive during the summer upwelling season (Broerse et al., 2000). This is the reason why we opted to not focus on the ecological indications of *U. sibogae* in our data.

6. You have correctly identified the impact of YTT as independent of the more regional climatic changes (interstadial-stadial) that occurred in the region.

**Reply:** Thank you for the comment. We believe that this is the case.

7. I should have emphasized more the potential dust fertilization that you briefly mention in the discussion, giving it the prominence it deserves. For instance, you could show a more detailed graphic of the major coccolithophore components shortly before (e.g., depths: 1845.6 to 1843.3), during (depths: 1843.1 to 1842.1), and shortly after (e.g., depths: 1842.0 to 1837.6) the event.

**Reply:** We explored other types of data visualizations to show this change in detail. However, we concluded to show the coccolithophore assemblage shifts in Figure 6 as a box plot, as we think that this way (1) statistical information is clearly shown and (2) such a plot is more accessible to readers outside of the micropaleontological field.

8. You could also have included (for the record) results for some of the less abundant species, like C. leptoporus, which interestingly did not show much change. For the other less abundant species, of course additional counts should have been made, particularly if your count of 300 includes small placoliths and/or F. profunda.

**Reply:** We appreciate this insight. We targeted 300 coccolith counts since we believe that this amount captures well the relative abundance variation of the major components of the coccolithophore assemblage (i.e., *G. oceanica*, small placoliths, *F. profunda*, *U. sibogae*, *H. carteri*), which already constitutes ~90% of the entire assemblage. For less abundant/rare species (i.e., ~2%) the 95% confidence interval is 1.5% based on a 300-specimen count. Thus, the detected changes in the relative abundance signal of the less abundant taxa are almost always within the statistical uncertainty.

We also underscore that the Arabian Sea is quite unique as *G. oceanica* is more abundant than small placoliths (i.e., *E. huxleyi*) and/or *F. profunda* in modern (Andruleit et al., 2000; Broerse et al., 2000; Andruleit et al., 2003) and fossil (Rogalla and Andruleit, 2005) coccolithophore assemblages. Thus, we think that capturing the variations of the major components alone are sufficient in our work and avoids the always looming risk of overinterpretation.

Reviewer #2 Evaluations:

1. The manuscript "Coccolithophore assemblage composition during the Greenland Interstadial–Stadial 20 transition and their response to the Youngest Toba Tuff (YTT) supereruption ~74,000 years ago in the northeastern Arabian Sea" by Guballa et al. presents a high-resolution dataset aimed at enhancing the understanding of coccolithophores during the stadial/interstadial transition and the YTT supereruption. Statistical and signal analyses are also used to elucidate and improve the primary relationships between coccolithophores and environmental factors.
The authors describe the data in great detail and illustrate their findings with an adequate number of high-quality figures. The references are up-to-date. I fully agree with the authors' conclusion that “the eruption was not the trigger for the GI-20 to GS-20 transition.” All my comments and suggestions are intended to improve the quality of the paper, which needs clarification in some parts of the text.

**Reply:** We thank Reviewer #2 for the positive comments on our work. We address their concerns below.

2. Line 22: “annually resolved” – The mean resolution is 7.85 years, and for the first 60 samples (before YTT), it is 7.9 years. I strongly suggest using “decadal resolved” not only in line 22 but throughout the text, particularly in the “Materials and Methods” section.

**Reply:** Thank you for the comment. We now revise “nearly annually resolved” to “temporal resolution from 2 to 21 years” to clarify the time resolution of our samples.

3. “Spectral analyses”: This is a critical point. The authors, using “redfit,” find a ~20-year periodicity in the first 60 samples (before YTT), but do not check (e.g., wavelet analyses) if this periodicity is consistently present throughout the interval. I have performed some checks: interpolating samples at 7.9 years (no artificial decreases were observed) and performing wavelet analysis, the ~20-year periodicity is barely visible only in the first 10 samples of the interval. Consequently, I think that this 20-year periodicity is negligible and useless (probably a multiple of the 11-year periodicity but impossible to check due to the “low” resolution).

**Reply:** We thank reviewer 2 for this important comment. We initially did a wavelet analysis but decided to use the “redfit” function in our data to avoid the problem of linear interpolation that artificially decreases the spectral power of higher frequency components (Schulz and Statteger, 1997; Schulz and Mudelsee, 2002). Since wavelet analysis require interpolation between data points to achieve equidistant sampling, this method decreases frequency resolution and the ~20-year band is suppressed as noticed by Reviewer #2. Nevertheless, we now provide additional frequency analyses (i.e., multitaper spectral analysis (MTM), wavelet analysis) in our Supporting Information as S3 and S4 Figs, which we think strengthens our argument for a multidecadal cycle at the 20–30-year band.

We first test whether MTM analysis (Thomson, 1982) on linearly interpolated data will show similar results to our “redfit” analysis. Indeed, we also find the 20 and 30-year bands using MTM (Figure 1) although the spectral power of the small placoliths seem to be weakened and a 30–50-year band is identified in *G. oceanica* (Figure 1A) and *F. profunda* (Figure 1D). More importantly, we did not detect significant power (i.e., 90% level) in the 70-year band in *G. oceanica* and the small placoliths (Figure 1A and 1B) but seems to be present in *U. sibogae* (Figure 1C).

Figure 1. Multitaper spectral analysis of the main coccolithophore species in Core SO130-289KL. (A) *G. oceanica*. (B) small placoliths consisting of *E. huxleyi* and *G. ericsonii*. (C) *U. sibogae*. (D) *F. profunda*. The black line shows the spectrum for each species. Brown, orange, and red lines represent the 90%, 95%, and 99% confidence limits, respectively. The yellow rectangles highlight bands exceeding the 90% confidence limit. Yellow rectangles with asterisks show bands that are very close to the 90% confidence limit. The horizontal line in (B) shows the bandwidth (BW) resolution.

Wavelet analysis (Torrence and Compo, 1998; Grinsted et al., 2004) on the interpolated dataset does not reveal a continuous power in the 20-year band which confirms the interpretation of Reviewer #2 (Figure 2). However, the suppression of the 20–year band might be the result of linear interpolation. Furthermore, the result (Figure 2, B and C) does not show significant power at the 70-year band (i.e., the 70-year band is within the gray shaded areas where results should be interpreted with caution).

Figure 2. Wavelet analysis of the major coccolithophore species in core SO130-289KL. A. *G. oceanica*. B. small placoliths consisting of *E. huxleyi* and *G. ericsonii*. C. *U. sibogae*. D. *F. profunda*. The heavy black contour lines denote the 95% significance level. Gray shaded regions show the cone of influence, where edge effects become important and thus should be interpreted with caution (i.e., the calculation of the spectral variance lies outside of the data boundaries).

4. Conversely, applying the Ensemble Empirical Mode Decomposition algorithm, I have found very interesting results in the IMF. Notably, there is a ~70-year periodicity in G. oceanica and E. huxleyi, showing a continuous anti-correlation (in line with their respective ecological preferences) in cross-wavelet analyses. I strongly suggest performing new signal analyses, especially since the authors noticed the effect of the 70-year periodicity (line 330) but did not fully explore the potential of this signal.

**Reply:**

We thank Reviewer #2 for leading us to the Ensemble Empirical Mode Decomposition (EEMD) method of signal analysis (Wu and Huang, 2009); we now added the analysis to our Supporting Information as S5 and S6 Figs for all major coccolithophore species. We used the “eemd” function found in the “Rlibeemd” package of Luukko et al. (2016) to perform the analysis. Since the EEMD is data-adaptive and does not rely on prior interpolation (Wu and Huang, 2009), we opted to perform the analysis on both raw and interpolated relative abundance data.

Indeed, the results show a multidecadal cyclicity in the second intrinsic mode function (IMF2,~70 years) in both *G. oceanica* and small placoliths that appears anticorrelated (Figure 3).

Figure 3. The second intrinsic mode function (IMF) extracted from the relative abundance of *G. oceanica* (blue) and small placoliths (orange). Data were interpolated at 7.9-year time steps prior to EEMD analysis.

We place the EEMD results into context by adding the second IMF (i.e., 70 years; the multidecadal cyclicity) with the residual (i.e., the monotonic function serving as the trend) to be able to compare with the relative abundance data (Figure 4):

Figure 4. The relative abundance data (thin lines) and the second IMF added with the residual (i.e., monotonic trend) (heavy lines) of *G. oceanica* (blue) and small placoliths (orange). This step was done to place the EEMD results in context with the relative abundance data. Note that the multidecadal cyclicity in the small placoliths seen in Figure 2 is muted.

We find that the cyclicities found in the small placoliths are suppressed relative to *G. oceanica* between 74250 to 73950 years ago, suggesting that a “continuous anti-correlation” in the 70-year band does not seem to be a consistently prominent feature (Figure 3).

More importantly, the MTM and wavelet analyses do not support the presence of a significant 70-year feature in *G. oceanica* and the small placolith data (Figures 1A and 1B, Figures 2A and 2B), which agree with our results using “redfit”. Instead, the 20–30-year band appears more consistently regardless of the frequency analysis method used.

We also underscore the lack of consistent presence of the 70-year cycle in other highly temporally resolved proxy records analyzed at the same time interval as our coccolithophore data in the region (Figure 5 below; now added as S7 Fig in our Supporting Information):

Figure 5. Spectral analyses of highly temporally resolved proxy records using the "redfit" (A-C) and multitaper (D-F) methods. A and D: L* reflectance record of Deplazes et al. (2013). B and E: the NGRIP δ^18^O record of Svensson et al. (2013). C and F: the speleothem δ^18^O record of Du et al. (2019). The black line shows the spectrum for each species. Brown, orange, and red lines represent the 90%, 95%, and 99% confidence limits, respectively. The yellow rectangles highlight bands exceeding the 90% confidence limit. Yellow rectangles with asterisks show bands that are very close to the 90% confidence limit. The horizontal line in (F) shows the bandwidth (BW) resolution for the multitaper analysis.

For instance, the ultra high resolved L* reflectance record of Deplazes et al. (2013) does not show a ~70-year band using “redfit” (Figure 5A) but seems to be present using MTM (Figure 5D). However, significant 16–19-year bands are present in both cases (Figures 5A and 5D). Furthermore, the North Greenland Ice Core Project (NGRIP) δ^18^O of Svensson et al. (2013) (Figures 5B and 5E) and the speleothem δ^18^O record of Du et al. (2019) (Figures 5B and 5E) also do not show such low frequency cycles. Remarkably, the NGRIP record consistently shows spectral power at the 15–30-year band consistent with the L* reflectance (Figures 5A and 5D) and our coccolithophore record (Figure 1).

Therefore, although the 70-year cyclicity as suggested by Reviewer #2 is a very interesting observation, it is not corroborated by any other proxy record nor statistical analysis that such a cyclicity exists in our record. Perhaps more temporally resolved records might shed light on the nature of this cyclicity.

5. Line 126: “using coccolithophore species that were consistently present” – Please specify the taxa.

**Reply:** Thank you for this comment. We wrote the sentence in the method section since we are considering the specific species list as a result. We fear that stating the species without any context of the results may cause some confusion for the reader. Therefore, we opt to not state the taxa at this point. However, we are happy to revise these lines at the recommendation of the editor.

6. Line 384: “the interpretation of the abundance changes in H. carteri …..” – In my opinion, the authors report interesting data on H. carteri. I suggest referencing Bonomo et al., Marine Micropaleontology, <https://doi.org/10.1016/j.marmicro.2021.101995> (see station 25). This paper reports (living coccoliths) an interesting and comparable result for H. carteri. Notably, the abundance of H. carteri (inside the mini slump interval) vs. small placoliths (E. huxleyi) shows comparable results, indicating that the coccolithophore assemblages exhibited an inversion of dominance, characterized by low E. huxleyi vs. relatively high abundance of H. carteri.

**Reply:** Thank you for the comment and the suggested reference. Bonomo et al. (2021) reported a maximum in the cell abundance (cells/l) of *H. carteri* between 25–75 m water depth in the Ligurian Sea (northwestern Italy) and exceeds the cell abundance of *E. huxleyi*. Their conclusion that *H. carteri* is an opportunistic species during times of high turbidity and high productivity do match our data right after the YTT eruption.

That said, stating an “inversion of dominance” may be misleading since the relative abundance of the small placoliths is still higher compared to *H. carteri* in the YTT interval.

7. Line 453 “Interdecadal cycles in the coccolithophore record during GI-20”: This chapter must be completely revised based on new signal analyses (see above) and consequently, the “Conclusion” chapter.

**Reply:** We thank Reviewer #2 for pointing us to additional frequency analyses that helped us improve our work. Based on our reanalysis (i.e., Points #3 and #4 above), we decided to adhere to our original results and interpretations for these sections.

However, we have updated the section “Interdecadal cycles in the coccolithophore record during GI-20” to reflect the additional analyses conducted on our data, the ~70-year periodicity that resulted from the EEMD analysis (added text are underlined):

“Further insights on the potential factors affecting the coccolithophore assemblage in our record may be revealed by spectral analysis. Spectral analysis of the coccolithophore record before the YTT eruption indicates the presence of an interdecadal band (~20–22 and 29 years) using the redfit, a method designed for unevenly spaced data (Fig 3). We also implemented additional frequency analysis on linearly interpolated data (S1 Text) that revealed broadly comparable results (S3 Fig) although these frequencies are significant in a particular time frame (S4 Fig). There also seems to be a lower frequency band in our data (i.e., 60–70-year cyclicity) (S5 and S6 Figs) but was not consistently detected (Fig 3 and S3 and S4 Figs). Thus, we also investigated whether other highly temporally resolved proxy records show frequencies at or near these interdecadal bands.

Indeed, the L* reflectance record [38] also show broadly comparable cyclicities at the 16–20-year band (S7A and S7D Figs). We also detected significant bands at 29–30 years, 19 years, and 15–16 years in the North Greenland Ice Core Project (NGRIP) δ^18^O record (S7B and S7E Figs) [88]. On the other hand, the power spectrum of the speleothem δ^18^O record [27] appears more complex and does not show a consistently present statistically significant band (S7C and S7F Figs). Moreover, these records do not show a consistent 60–70-year band (S7 Fig) but appear to exhibit a 20–30-year cyclicity similar to our data (Fig 3). The detection of broadly comparable bands in ice [88], marine ([38] and this study) and, possibly, continental records [27] in the 20–30-year band (Fig 3 and S3 and S7 Figs) suggests that a global-scale mechanism might have driven the observed cycles although the exact process currently remains uncertain.”

That said, we added in our Supporting Information a concise text outlining the other frequency analyses performed to our data:

“S1 Text. Additional frequency analyses using linearly interpolated data.

In addition to the “redfit” function, several other methods of frequency analyses were performed on the data set such as multitaper spectral analysis (MTM; Thomson, 1982) and wavelet analysis (Torrence and Compo, 1998; Grinsted et al., 2004). To perform these analyses, the relative abundance data were linearly interpolated at 7.9-year time steps (i.e., mean time interval). We then used the Acycle software (Li et al., 2019) to conduct the frequency analyses. We used a time-bandwidth product of 2 for the MTM analysis. Lastly, we implemented ensemble empirical mode decomposition (EEMD) analysis (Wu and Huang, 2009)—a data-adaptive approach that does not require stationarity or linearity in extracting intrinsic oscillations (i.e., intrinsic mode functions) in the data. The EEMD analysis was done using the “rlibeemd” package in R using the default settings (Luukko et al., 2016).

The MTM spectral analysis shows broadly comparable results with the “redfit” analysis (S3 Fig) although wavelet spectra indicate that these frequencies are significant in a particular time frame (S4 Fig). On the other hand, signal decomposition using the EEMD revealed a ~60–70-year cyclicity in *G. oceanica* and the small placoliths that seem to be anticorrelated (S5 and S6 Figs). However, such a cyclicity was not detected from “redfit” (Fig 3), MTM (S3 Fig), and wavelet analyses (S4 Fig).”

References cited in the responses to reviewers:

Aitchison J. Principles of compositional data analysis. In: Anderson TW, Fang KT, Olkin I, editors. Multivariate analysis and its applications. Hayward, California: Institute of Mathematical Sciences; 1994. pp. 73–81. Available: https://doi.org/10.1214/lnms/1215463786

Andruleit H, von Rad U, Brans A, Ittekkot V. Coccolithophore fluxes from sediment traps in the northeastern Arabian Sea off Pakistan. Mar Micropaleontol. 2000;38: 285–308. doi:10.1016/S0377-8398(00)00007-4

Andruleit H, Stäger S, Rogalla U, Čepek P. Living coccolithophores in the northern Arabian Sea: ecological tolerances and environmental control. Mar Micropaleontol. 2003;49: 157–181. doi:10.1016/S0377-8398(03)00049-5

Bonomo S, Schroeder K, Cascella A, Alberico I, Lirer F. Living coccolithophore communities in the central Mediterranean Sea (Summer 2016): Relations between ecology and oceanography. Mar Micropaleontol. 2021;165: 101995. doi:10.1016/j.marmicro.2021.101995

Broerse ATC, Brummer G-JA, Hinte JEV. Coccolithophore export production in response to monsoonal upwelling off Somalia (northwestern Indian Ocean). Deep Sea Res Part II Top Stud Oceanogr. 2000;47: 2179–2205. doi:10.1016/S0967-0645(00)00021-7

Deplazes G, Lückge A, Peterson LC, Timmermann A, Hamann Y, Hughen KA, et al. Total reflectance of sediment core SO130-289KL. PANGAEA; 2013. doi:10.1594/PANGAEA.815851

Du W, Cheng H, Xu Y, Yang X, Zhang P, Sha L, et al. Timing and structure of the weak Asian Monsoon event about 73,000 years ago. Quat Geochronol. 2019;53: 1–10. doi:10.1016/j.quageo.2019.05.002

Grinsted A, Moore JC, Jevrejeva S. Application of the cross wavelet transform and wavelet coherence to geophysical time series. Nonlinear Process Geophys. 2004;11: 561–566. doi:10.5194/npg-11-561-2004

Li M, Hinnov L, Kump L. Acycle: Time-series analysis software for paleoclimate research and education. Comput Geosci. 2019;127: 12–22. doi:10.1016/j.cageo.2019.02.011

Luukko PJJ, Helske J, Räsänen E. Introducing libeemd: a program package for performing the ensemble empirical mode decomposition. Comput Stat. 2016;31: 545–557. doi:10.1007/s00180-015-0603-9

Rogalla U, Andruleit H. Precessional forcing of coccolithophore assemblages in the northern Arabian Sea: Implications for monsoonal dynamics during the last 200,000 years. Mar Geol. 2005;217: 31–48. doi:10.1016/j.margeo.2005.02.028

Schulz M, Stattegger K. Spectrum: spectral analysis of unevenly spaced paleoclimatic time series. Comput Geosci. 1997;23: 929–945. doi:10.1016/S0098-3004(97)00087-3

Schulz M, Mudelsee M. REDFIT: estimating red-noise spectra directly from unevenly spaced paleoclimatic time series. Comput Geosci. 2002;28: 421–426. doi:10.1016/S0098-3004(01)00044-9

Svensson AM, Bigler M, Blunier T, Clausen HB, Dahl-Jensen D, Fischer H, et al. Direct linking of Greenland and Antarctic ice cores at the Toba eruption (74 ka BP). Clim Past. 2013;9: 749–766. doi:10.5194/cp-9-749-2013

Thomson DJ. Spectrum estimation and harmonic analysis. Proc IEEE. 1982;70: 1055–1096. doi:10.1109/PROC.1982.12433

Torrence C, Compo GP. A Practical Guide to Wavelet Analysis. Bull Am Meteorol Soc. 1998;79: 61–78. doi:10.1175/1520-0477(1998)079<0061:APGTWA>2.0.CO;2

Wu Z, Huang NE. Ensemble Empirical Mode Decomposition: A noise-assisted data analysis method. Adv Adapt Data Anal. 2009;01: 1–41. doi:10.1142/S1793536909000047
